# Chemical Constituents and Bioactivities of the Plant-Derived Fungus *Aspergillus fumigatus*

**DOI:** 10.3390/molecules29030649

**Published:** 2024-01-30

**Authors:** Zihuan Sang, Yanjiang Zhang, Kaidi Qiu, Yuting Zheng, Chen Chen, Li Xu, Jiaying Lai, Zhenxing Zou, Haibo Tan

**Affiliations:** 1 Hunan Key Laboratory of Diagnostic and Therapeutic Drug Rsearch for Chronic Diseases, Xiangya School of Pharmaceutical Sciences, Central South University, Changsha 410013, China; sangzihuan123@163.com (Z.S.); 217211011@csu.edu.cn (Y.Z.); chenchenpp09@163.com (C.C.); 217211012@csu.edu.cn (L.X.); 2 National Engineering Research Center of Navel Orange, Gannan Normal University, Ganzhou 341000, China; 3 Key Laboratory of South China Agricultural Plant Molecular Analysis and Genetic Improvement, Guangdong Provincial Key Laboratory of Applied Botany, South China Botanical Garden, Chinese Academy of Sciences, Guangzhou 510650, China; hnzhangyanjiang@163.com (Y.Z.); qiukaidi21@scbg.ac.cn (K.Q.); laijiaying22@mails.ucas.ac.cn (J.L.)

**Keywords:** sesquiterpenoid, *Aspergillus fumigatus*, MRSA, antibacterial activity

## Abstract

A new bergamotane sesquiterpenoid, named xylariterpenoid H (**1**), along with fourteen known compounds (**2**–**15**), were isolated from the crude extract of *Aspergillus fumigatus*, an endophytic fungus isolated from *Delphinium grandiflorum* L. Their structures were elucidated mainly by extensive analyses of NMR and MS spectroscopic data. In addition, the screening results of antibacterial and cytotoxic activities of compounds **1**–**15** showed that compound **4** displayed antibacterial activities against *Staphylococcus aureus* and MRSA (methicillin-resistant *S. aureus*) with an MIC value of 3.12 µg/mL.

## 1. Introduction

*Delphinium grandiflorum* L., a tremendously important species of the genus *Delphinium* belonging to the family Ranunculaceae, is widely distributed in China and India [1]. Previous studies have reported the isolation and identification of a variety of biologically meaningful natural compounds from *D. grandiflorum* L., including diterpene alkaloids, flavonoids, and phenolic acids [2,3]. However, there are no related reports on the chemical constituents of endophytic fungi of *D. grandiflorum* L., which, thus, successfully aroused our research interest. Therefore, our research group conducted phytochemical and biological activity screenings on endophytic fungi isolated from *D. grandiflorum* L., and finally selected *Aspergillus fumigatus* as the targeted research strain. 

**Figure 1:**
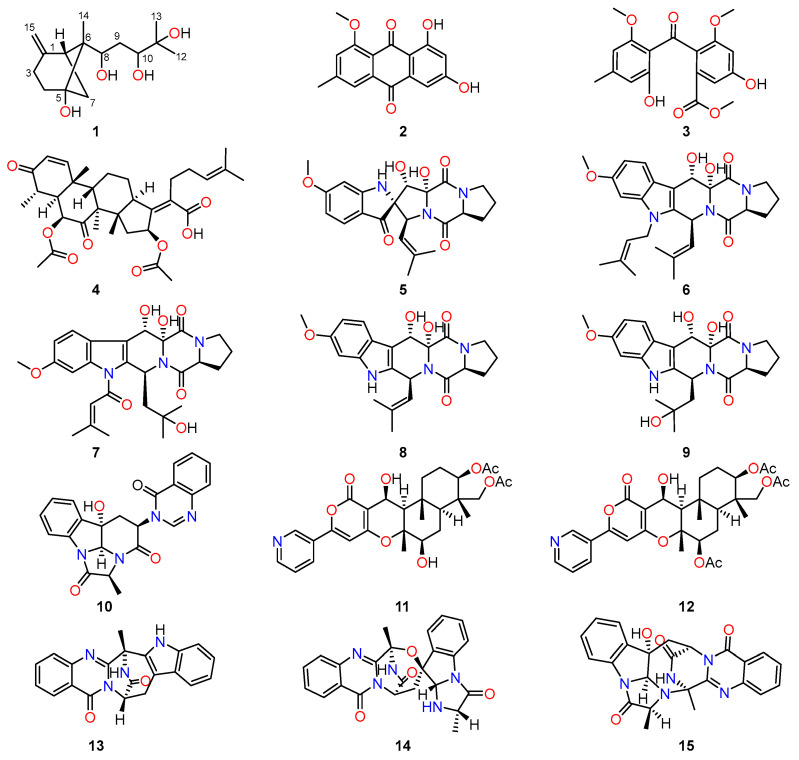
The chemical structures of compounds **1**–**15**.

The previous excellent phytochemical studies on endophytic fungi of *A. fumigatus* have established the presence of pyranones [4,5], terpenes [6,7], alkaloids [8], and thiophenols [9], most of which showed considerable biological activities such as antibacterial [10], anticancer [11], anti-inflammatory [12], and antioxidant activities [9]. In this study, a previously undescribed compound with the name of xylariterpenoid H (**1**), together with fourteen known compounds (**2**–**15**), was successfully isolated and identified from the endophytic fungus *A. fumigatus* by our research group (Figure 1). Moreover, the antibacterial and cytotoxic activities of these isolated compounds **1**–**15** were assayed, wherein compound **4** had been disclosed to display very significant antibacterial activities against *Staphylococcus aureus* and MRSA (methicillin-resistant *S. aureus*). Herein, the details of the extraction, purification, structure elucidation, and their biological evaluation are described.

## 2. Results and Discussion

### 2.1. Structure Characterization of Isolated Compounds

Compound **1** was isolated as a colorless oil with the chemical molecular formula of C_15_H_26_O_4_ deduced by HRESIMS (Figure S1) at *m*/*z* 293.1740, [M + Na]^+^ (calculated for 293.1729), accounting for three degrees of unsaturation. Its IR (KBr) spectrum (Figure S4) exhibited absorptions at 3375 cm^−1^ (hydroxy) and 1637 cm^−1^ (double bond). Analyses of the ^1^H NMR data (Figure S5 and Table 1) revealed the presence of three singlet methyl groups (*δ*_H_ 0.81 (3H, s, H-14), 1.20 (3H, s, H-12), and 1.25 (3H, s, H-13)), two terminal olefin proton signals (*δ*_H_ 4.63 (1H, brs, H-14a), 4.69 (1H, brs, H-14b)). Furthermore, its ^13^C-NMR and HSQC spectra (Figures S6 and S8) exhibited the signals of 15 carbon resonances, including three methyls (δC 10.5, 23.9, and 26.4), four methylenes (δC 25.2, 31.7, 33.5, and 36.1), three methines involving an olefinic carbon (δC 70.4, 74.9, and 107.9), and four nonprotonated carbons at δC 52.3, 73.2, 76.7, and 147.7. These results together with the molecular formula, suggested that compound **1** was most likely a sesquiterpenoid. Considering the three degrees of unsaturation in the molecule and the terminal olefin double bond accounting for one of the degrees of unsaturation, the remaining two degrees of hydrogen deficiency necessitated compound **1** should possess a bicyclic ring system.

**Figure 2:**
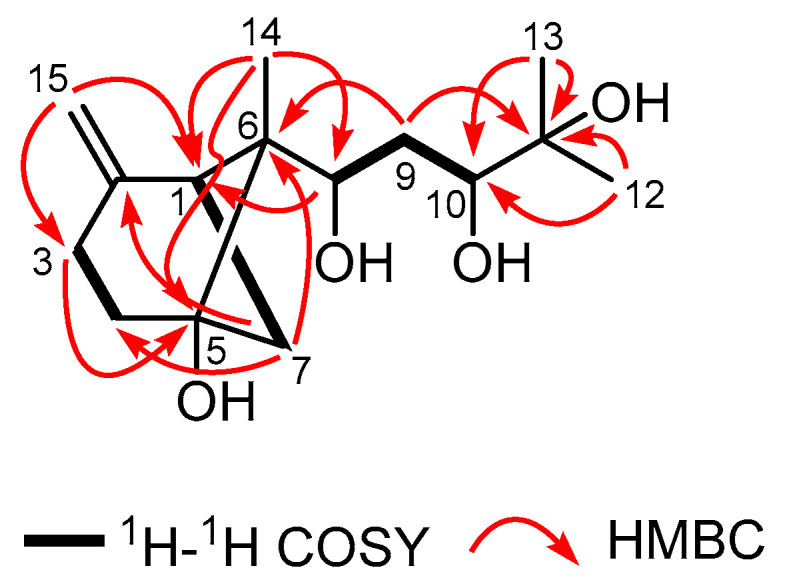
Key ^1^H-^1^H COSY and HMBC correlations of compound **1**.

In order to construct the bicyclic skeleton of compound **1**, the 2 D NMR spectra involving both to the HMBC and ^1^H-^1^H COSY (Figure 2) spectra were performed and elucidated. The HMBC spectrum (Figure S9) showed the cross peak from the terminal olefin proton H-15 (*δ*_H_ 4.69 and 4.63) to C-1 (*δ*_C_ 42.0), C-2 (*δ*_C_ 147.7), and C-3 (*δ*_C_ 25.2), from H-3 (*δ*_H_ 2.36 and 2.63) and H-7 (*δ*_H_ 1.91 and 2.47) to C-5 (*δ*_C_ 76.7). Along with the COSY correlations (Figure S7) of H-1 (*δ*_H_ 2.35) with H-7 and of H-3 with H-4 (*δ*_H_ 1.79 and 1.98) indicated the presence of a 4-methylene cyclohexanol ring in the molecule. Additionally, the HMBC correlations of H-14 (*δ*_H_ 0.82) with C-1 (*δ*_C_ 42.0), and C-5 (*δ*_C_ 76.7), of H-7 with C-4 and C-6 (*δ*_C_ 52.3) suggested that the presence of a 6-methylbicyclo [3.1.1] heptane skeleton. In addition, based on the ^1^H-^1^H COSY correlations from H-8 to H-10, the HMBC correlations of the methylene proton H-9 (*δ*_H_ 1.35) with the oxygenated carbon C-11 (*δ*_C_ 73.2) and of the methyl protons H-12 (*δ*_H_ 1.24) and H-13 (*δ*_H_ 0.82) with C-10 (*δ*_C_ 74.9) and C-11 suggested the presence of a 1,3,4-trihydroxyl-4-methylpent side chain in **1**. Finally, the linkage of the two moieties was secured by the HMBC correlations of H-9 (*δ*_H_ 1.35) with C-6 (*δ*_C_ 52.3) as well as of H-14 to C-8. On the basis of the above evidence, the planar structure of compound **1** was thus established, which suggested that compound **1** should be a new bergamotane sesquiterpene. This type of compound was once isolated from a deep-sea-derived fungus [13]. Following the naming of this type of compound by Niu et al., the name of compound 1 was determined to be xyloterpene H.

**Figure 3:**
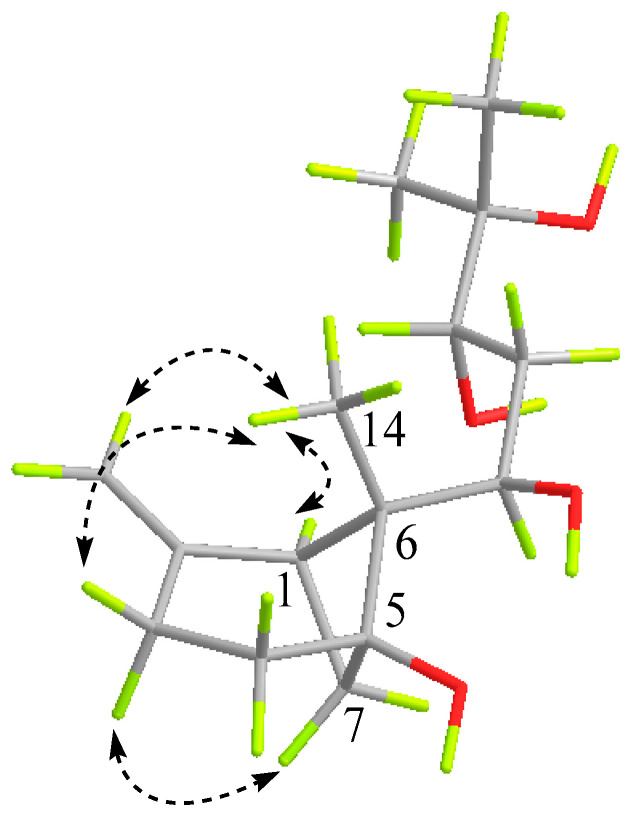
Key NOESY correlations of compound **1**.

The partial relative configuration of **1** was confirmed by the NOESY experiment (Figure S10 and Figure 3), based on the informative NOE correlations observed between H-3*α*/H-7*α,* which suggested that the two protons were cofacial and were arbitrarily assigned as *α*-orientation. The critical NOE interactions observed between H-3*β*/H_3_-14, H-3*β*/H_2_-15, and H-1/H_3_-14 indicated H-1 and H_3_-14 were oriented in the same direction. Then, the relative configuration of the cyclohexane ring and bridged cyclobutane ring were established.

However, the relative configuration of 1,3-dihydroxyl functionality for C-8 and C-10 positions in compound **1** was a failure to be determined. Although the mosher ester strategy towards the determination of the absolute configuration of this 1,3-dihydroxyl moiety was conducted, it provided a complex mixture, probably attributing to the presence of four free hydroxyl groups. The acetonide derivation of the 1,3-dihydroxyl moiety with acetone was also performed to establish the relative configuration, whereas it generated the acetonide product of C-10 and C-11 hydroxyls with low yield. Moreover, the ECD and ^13^C NMR calculations were also evidenced to be inefficient due to the existence of too many probable configurations caused by the two unestablished chiral centers. Therefore, the relative and absolute configurations of compound 1 had not been completely determined because of its limited amount and intractable structure characteristic in this study.

Notably, fourteen known compounds were also successfully isolated from the endophytic fungus *A. fumigatus*, and their structures were then identified as 1-methyl emodin (**2**) [14], monomethylsulochrin (**3**) [15], helvolic acid (**4**) [16], spiro-[5H,10H-dipyrrolo[1,2-a:1′,2′-d]pyrazine-2-(3H),2′-[2H]indole]-3′,5,10(1′H)-trione (**5**) [17], fumitremorgin B (**6**) [18], asperfumigatin (**7**) [10], 12,13-dihydroxyfumitremorgin C (**8**) [19], verruculogen TR-2 (**9**) [20], chaetominine (**10**) [21], 7-deacetylpyripyropene A (**11**) [22], pyripyropene A (**12**) [22,23], fumiquinazoline J (**13**) [24], fumiquinazoline C (**14**) [25], and fumiquinazoline D (**15**) [25] by comparing their spectroscopic data (Figures S11–S38) with those of the reported literatures. The structures of these known compounds are shown in Figure 1.

### 2.2. Antibacterial Activity

All of the isolated compounds were evaluated for their antibacterial activities against the Gram-positive bacteria *S. aureus* and MRSA by the microbroth dilution method [26]. As a result (Table S1 and Figure S39), among these tested compounds, helvolic acid (**4**) exhibited potent antibacterial activity against *S. aureus* and MRSA with MIC values of 3.12 μg/mL. Moreover, compound **3** exhibited modest antibacterial activity against *S. aureus* and MRSA with MIC values of 20 μg/mL. Unfortunately, the MIC values of compound **1** for all tested strains exceeded 100 μg/mL, and other compounds did not show any significant antibacterial activities. In order to evaluate the effect of helvolic acid on other strains, vancomycin-resistant *Enterococci* (VRE), vancomycin-sensitive *Enterococci* (VSE), and Gram-negative bacterium *Shigella dysenteriae* were chosen to perform the antibacterial experiments, and the biological screening results illustrated that the MIC values for helvolic acid towards these tested strains were 12.5, 25, and 100 μg/mL, respectively (Table S2). The results collectively pointed to helvolic acid (**4**) showing broad antibacterial spectrum with significant activities for the development of antibacterial innovative drugs.

### 2.3. Cytotoxic Activity

In addition, the antiproliferative effects of the isolated compounds **1**–**15** were further evaluated by a panel of human cancer cell lines, including Hela, HepG2, and A549. However, none of them showed any noticeable cytotoxic activity, even at the concentration of 50 μM. Among them, the inhibitory rates of compound **1** against A549, Hela, and HepG2 at 50 μM were 25.46%, 31.90%, and 28.55%, respectively; the inhibitory rates of compound **4** against A549, Hela, and HepG2 at 50 μM were 64.81%, 30.54%, and 69.12%, respectively. The intriguing result of neglectable cytotoxicity for helvolic acid (**4**) tentatively suggested that helvolic acid (**4**) could exhibit significant biological activities against a broad panel of bacteria with potent selectivity, which thus strongly indicated that helvolic acid might serve as a promising lead compound for the further development of anti-infective innovative drugs with limited cytotoxicity in future.

### 2.4. Discussion

The microbial community can be described as a “bio-diversified tropical rainforest”. It contains a large number of biologically active substances, which are a series of tremendously important sources of new drugs and active leads [27]. The helvolic acid isolated from *A. fumigatus* belongs to the fusidane-type antibiotics, and it has remarkable antibacterial activity against Gram-positive bacteria, especially *S. aureus*. Fusidane-type antibiotics belong to the only type of fungal triterpene with proterpene alcohol as the mother core, representing the only triterpene-derived antibiotic class [28], and they have been known for nearly 80 years [29]. The two representative drugs are cephalosporin P1 and fusidic acid, of which fusidic acid has been widely used in clinical therapeutics [30,31].

Currently, commercially available antibiotics with different mechanisms of action are experiencing resistance crises to varying degrees. However, the rate of development of bacterial resistance is much faster than the rate of antibiotic development, and resistant strains towards all of the usually-used antibiotics have been clinically detected. Therefore, the continuation of exploring new drug targets to meet the challenge of the antibiotic crisis is still extremely appealing. To our surprise, fusidane-type antibiotic helvolic acid (**4**) exhibited potent antibacterial activity against MRSA with a MIC value of 3.12 μg/mL. Notably, fusidane-type antibiotics are the only known antibiotics that selectively target bacteria elongation factor G (EF-G) [32] to show potent bacteriostatic and bactericidal effects [33]. The specific antibacterial mechanism of the fusidane-type antibiotics logically indicates that helvolic acid (**4**) might lead to little antibacterial cross-resistance in comparison with other commonly used antibiotics.

Helvolic acid (**4**) possesses intriguing structural features and excellent biological activity, which aroused an emerging new interest among chemists and biologists regarding the growing threat of antibiotic resistance. After the identification of the helvolic acid biosynthetic gene cluster (BGC) of *A. fumigatus* Af 293 in 2009, biosynthetic research on fusidane-type antibiotics has developed vigorously [34,35], and the biosynthetic pathway of helvolic acid has been fully proposed so far [36]. In this study, helvolic acid (**4**) demonstrated potent activity against bacterial pathogens, suggesting it was responsible for the antimicrobial activity initially observed in the crude extract of *A. fumigatus*. In future, the biosynthetic synthesis with epigenetic regulation of *A. fumigatus* towards the abundant generation of helvolic acid (**4**) is also appealed for the devolvement of *A. fumigatus* as a promising antibacterial biological agent.

Alkaloid molecules contain an N atom and have great structural diversity. Depending on the function of the amine, alkaloids can act as either a hydrogen-receptor or a hydrogen-donor for hydrogen bonding, which is crucial for the drug to exert its function [37]. It is worth mentioning that we have isolated multiple different types of alkaloids from *A. fumigatus*, including indole diketopiperazine alkaloids, quinazoline alkaloids, and pyridine alkaloids. According to literature reports, indole diketopiperazine alkaloids (IDAs) have significant pharmacological activities such as antimicrobial [38,39,40,41,42], antiviral [43,44,45,46], anticancer [47,48,49], immunomodulatory [50], antioxidant [51], and insecticidal activities [52]. Therefore, they may have promising potential to be used in drugs and/or serve as lead structures for drug development. Meanwhile, quinazoline alkaloids (QAs) as a series of heterocyclic compounds with nitrogen are one of the most significant heterocyclic motifs with diverse chemical reactivities and biological applications [53,54]. Especially, their derivatives play a crucial role in medicinal chemistry, evident in the chemical makeup of a wide range of FDA approved medications, clinical candidates, and bioactive compounds [55]. Unfortunately, none of the isolated alkaloids did not show any obvious antibacterial or cytotoxic activity in our preliminary pharmacological activity experiments. In future, the research efforts on the structural and pharmacological diversities of IDAs and QAs from the endophytic fungi *A. fumigatus* were still required to disclose their potent pharmacological applications.

During the isolation of *A. fumigatus*, we isolated one new compound and fourteen old compounds. By reviewing the literature, we found that changing some experimental conditions may be able to obtain more novel secondary metabolites. For example, adding 3-hydroxytyrosol, a new signaling molecule in fungi that can regulate biofilm growth, to the culture medium promoted the biotransformation process [56]. Inoculating medicinal plants with arbuscular mycorrhizal fungi (AMF) represents an alternative approach to enhance the quality and quantity of secondary metabolites. AMF can form endophytes or symbiotic relationships with numerous microorganisms in different parts of the plant. Subsequently, they influence the production of secondary metabolites by indirectly stimulating the biosynthetic pathways of these compounds [57].

Moreover, the strain of Aspergillus used in this experiment possesses enzymes such as cytochrome P450s (CYPs) with broad substrate specificity. *A. fumigatus* and its enzymes exhibit significant potential in biotransformation, bioremediation of environmental contaminants, and the biocatalytic production of essential compounds [58].

## 3. Materials and Methods

### 3.1. General Experimental Procedures

Optical rotations were measured on an MCP-500 spectropolarimeter (Anton Paar, Graz, Austria). UV spectra and ECD spectra were acquired on a UV-2600 spectrophotometer (Shimadzu, Kyoto, Japan). IR spectra were obtained on an Affinity-1 spectrometer (Shimadzu, Kyoto, Japan) using KBr discs. NMR spectra were recorded on a Bruker Avance-500 spectrometer (Bruker, Fällanden, Switzerland) using residual solvent signals as references. HRESIMS data were acquired by the Thermo MAT95XP spectrometer (Thermo Fisher Scientific, Bremen, Germany). Silica gel (80–100, 100–200, and 200–300 mesh, Qingdao Puke Parting Materials Co., Ltd., Qingdao, China) used for flash column chromatography was purchased commercially. The TLC analysis was carried out using commercially available silica gel plates (Qingdao Puke Parting Materials Co., Ltd., Qingdao, China). A Hitachi Primaide (Hitachi Instruments (Dalian) Co., Ltd., Dalian, China) equipped with a diode array detector (DAD) using a preparative YMC ODS C18 column (20 mm × 250 mm, 5 μm) was used for semipreparative HPLC separation. All solvents were analytical grade and used without further purification (Guangzhou Chemical Regents Company, Ltd., Guangzhou, China).

### 3.2. Fungal Material and Fermentation

The fungus *A. fumigatus* was isolated from *Delphinium grandiflorum* L., which was collected from the Aba Tibetan Autonomous Prefecture in July 2021. The plant species was authenticated on the basis of morphological characteristics and comparison with specimens, and the fungal species was authenticated on the basis of morphological characteristics and ITS DNA sequence data (GenBank: No. MT529485.1). The strain was preserved at Xiangya School of Pharmaceutical Sciences, Central South University, Changsha. The strain was cultured on potato dextrose agar (PDA) at 28 °C for 5 days containing 200 g/L of potato, 20 g/L of glucose, 3 g/L of KH_2_PO_4_, 1.5 g/L of MgSO_4_•7H_2_O, and 10 mg/L of vitamin B_1_ in distilled water. Then, a quarter of the agar with fungal colony was added to a conical flask (500 mL) with 250 mL of potato dextrose liquid medium, and the flask was incubated on a rotary shaker at 28 °C and 140 rpm for 5 days to prepare seed culture. Agar plugs were inoculated into 80 Erlenmeyer flasks (1 L) that were previously sterilized by autoclaving, with each containing 250 g of rice and 200 mL of distilled water. All flasks were incubated at 28 °C for 30 days.

### 3.3. Extraction and Isolation

The fermented rice substrate was extracted 3 times with EtOAc at room temperature, and the solvent was evaporated under vacuum to yield a total extract (50.9 g). The crude extract was subjected to silica gel column chromatography eluting with petroleum ether and EtOAc (100:1 to 1:1, *v*/*v*) as well as EtOAc and MeOH (1:1 to 1:5, *v*/*v*) to afford six main fractions (Fr. 1–Fr. 6).

Fraction 3 (7.5 g) was fractionated by an ODS column chromatography eluted with a gradient of MeOH-H_2_O (*v*/*v*, 40:60 → 100:0) to obtain seven subfractions (Fr.3-1 to Fr.3-7). Fr.3-2 (1.5 g) was separated by Sephadex LH-20 CC and eluted with CH_2_Cl_2_-MeOH (*v*/*v*, 1:3) to afford five subfractions (Fr.3-2-1 to Fr.3-2-5). Fr.3-2-3 was further fractionated by using semipreparative HPLC (MeCN-H_2_O, 50:50, v = 2.0 mL/min) to give compound **9** (21.0 mg, t_R_ = 8.0 min) and compound **10** (6.7 mg, t_R_ = 10.0 min). Fr.3-2-4 was isolated on silica gel and eluted with petroleum ether-EtOAc gradient (*v*/*v*, 100:1 → 1:2) to obtain six sub-fractions (Fr.3-2-4-1 to Fr.3-2-4-6). Fr.3-2-4-3 was further purified by silica gel, eluting with CH_2_Cl_2_-MeOH (*v*/*v*, 1:0 → 20:1) to afford compound **5** (4.2 mg). Fr.3-2-4-6 was further purified by silica gel, eluting with ether-EtOAc (*v*/*v*, 1:2 → 1:5) to afford compound **1** (4.9 mg).

Fr.3-3 (1.1 g) was separated by Sephadex LH-20 CC and eluted with CH_2_Cl_2_-MeOH (*v*/*v*, 1:3) to afford six subfractions (Fr.3-3-1 to Fr.3-3-6). Fr.3-3-3 was further fractionated by using semipreparative HPLC (MeCN-H_2_O, 60:40, v = 2.0 mL/min) to give compound **11** (4.2 mg, t_R_ = 12.0 min). Fr.3-3-5 was further fractionated by using semipreparative HPLC (MeCN-H_2_O, 65:35, v = 2.0 mL/min) to give compound **8** (3.7 mg, t_R_ = 22.0 min). Fr.3-4 (0.7 g) was separated by Sephadex LH-20 CC and eluted with CH_2_Cl_2_-MeOH (*v*/*v*, 1:3) to afford four subfractions (Fr.3-4-1 to Fr.3-4-4). Fr.3-4-1 was further fractionated by using silica gel, eluting with CH_2_Cl_2_-MeOH (*v*/*v*, 1:0 → 50:1) to afford compound **12** (11.2 mg). Fr.3-4-2 was purified by silica gel and eluted with CH_2_Cl_2_-MeOH gradient (*v*/*v*, 100:1 → 10:1) to obtain compound **7** (7.5 mg). Fr.3-5 (1.6 g) was separated by Sephadex LH-20 CC and eluted with CH_2_Cl_2_-MeOH (*v*/*v*, 1:3) to afford five subfractions (Fr.3-5-1 to Fr.3-5-5). Fr.3-5-4 was isolated on silica gel and eluted with petroleum ether-EtOAc gradient (*v*/*v*, 100:1 → 1:2) to obtain compound **6** (25.5 mg).

Fraction 2 (8.2 g) was fractionated by an ODS column chromatography eluted with a gradient of MeOH-H_2_O (*v*/*v*, 40:60 → 100:0) to obtain six subfractions (Fr.2-1 to Fr.2-6). Fr.2-2 (1.5 g) was separated by Sephadex LH-20 CC and eluted with CH_2_Cl_2_-MeOH (*v*/*v*, 1:3) to afford four subfractions (Fr.2-2-1 to Fr.2-2-4). Fr.2-2-2 was purified by silica gel and eluted with CH_2_Cl_2_-MeOH gradient (*v*/*v*, 100:1 → 10:1) to obtain compound **14** (79.0 mg). Fr.2-2-6 was further fractionated by using semipreparative HPLC (MeCN-H_2_O, 60:40, v = 2.0 mL/min) to give compound **15** (2.8 mg, t_R_ = 14.0 min). Fr.2-3 (0.6 g) was separated by Sephadex LH-20 CC and eluted with CH_2_Cl_2_-MeOH (*v*/*v*, 1:3) to afford five subfractions (Fr.2-3-1 to Fr.2-3-5). Fr.2-3-5 was purified by silica gel and eluted with ether-EtOAc (*v*/*v*, 10:1 → 1:1) to obtain compounds **2** (4.4 mg) and **13** (11.8 mg). In addition, a white solid was precipitated in Fr.2-3 to give compound **3** (55.0 mg), and a large amount of white solid was precipitated in Fr.2-5 to give compound **4** (808.0 mg).

Xylariterpenoid H (**1**): colorless oil; [α]^25^_D_ −0.15 (c 0.1, MeOH); ECD (MeOH) λmax (Δε): 200 (+11.84) nm; UV (MeOH) *λ*max (log ε): 200 (1.92) nm; IR (KBr): 3853, 3375, 2922, 2852, 1734, 1637, 1465, 1186, 962, 721, 518 cm^−1^, ^1^H (500 MHz) and ^13^C (125 MHz) NMR data see Table 1. HRESIMS: *m*/*z* 293.1740 [M + Na]^+^ (calculated for C_15_H_26_O_4_Na, 293.1729).

### 3.4. Cytotoxic Activity Assay

Cytotoxic viability was determined by using the SRB method [59]. The cell lines (Hela, HepG2, and A549) were cultured in RPMI-1640 medium with 10% fetal bovine serum at 37 °C. The suspended cells were seeded in 96-well plates at a density of 3 × 10^4^ cells/mL in an incubator under an atmosphere of 5% CO_2_ at 37 °C for 24 h. Then, 20 μL of various concentrations of compounds were added and further incubated for 72 h. After that, the cell monolayers were fixed by 50% (*wt*/*v*) trichloroacetic acid (50 μL) and stained for 30 min by 0.4% (*wt*/*v*) SRB, which was dissolved in 1% acetic acid. The unbound dye was removed by washing repeatedly with 1% acetic acid, and the resulting cells were then dissolved the protein-bound dye in 10 mM Tris base solution (200 μL), and the absorbance was measured at 570 nm. Cisplatin was used as a positive control possessing potent cytotoxic activity. All data were obtained in triplicate and are presented as means ± S.D.

### 3.5. Antibacterial Assay

All isolated compounds were evaluated against bacteria strains embodying *S. aureus* (CMCC 26003), MRSA (NCTC 10442), *Escherichia coli* (ATCC 8739), VRE (No. 151458137), and VSE (No. 160119481), all of which were obtained from Guangdong Microbiology Culture Center (Guangzhou, China). MIC values were determined by the methodology of microbroth dilution in Mueller–Hinton broth medium (MHB) according to CLSI guidelines; the positive control was vancomycin or polymyxin B. Briefly, 20 μL tested compounds with a concentration of 1 mg/mL was added to 180 μL bacterial liquid, and the method of double dilution was adopted in 96-well plates. The lowest concentration of the drug preventing visible growth of the pathogen was taken as the MIC.

## 4. Conclusions

In summary, this study performed a comprehensive chemical investigation on the bioactive natural product of the endophytic fungi *A. fumigatus* isolated from *Delphinium grandiflorum* L., and it has resulted in the successful isolation and structure identification of an undescribed compound xylariterpenoid H (**1**) together with fourteen known compounds (**2**–**15**). The biological activity screening of these isolates revealed that helvolic acid (**4**) exhibited significantly potent antibacterial activities against *S. aureus* and MRSA, which were comparable to the positive control vancomycin without any significant cytotoxicity, revealing tremendous promise in the development of innovative anti-infective drugs. These findings not only disclose the biological chemical constitute of *A. fumigatus* but also note the further development potential of *Delphinium grandiflorum* L. Furthermore, the antibacterial mechanism experiments towards the bioactive lead compound helvolic acid (**4**) are now underway and will be revealed in due course.

## Figures and Tables

**Table 1:** ^1^H (500 MHz) and ^13^C (125 MHz) NMR data for compound **1** in CDCl_3_.

Position	*δ*_C_	*δ*_H_ (*J* in Hz)
1	42.0, CH	2.35, d (6.9)
2	147.7, C	
3	25.2, CH_2_	2.35, m 2.63, m
4	31.7, CH_2_	1.79, m 1.98, m
5	77.2, C	
6	52.3, C	
7	36.1, CH_2_	1.89, d (10.0) 2.51, dd (10.0, 6.9)
8	70.4, CH	4.66, m
9	33.5, CH_2_	1.35, m 1.60, ddd (13.4, 10.4, 2.7)
10	74.9, CH	3.72, m
11	73.2, C	
12	23.9, CH_3_	1.20, s
13	26.4, CH_3_	1.24, s
14	10.5, CH_3_	0.82, s
15	107.9, CH_2_	4.62, brs 4.68, brs

## Data Availability

All the data, including HRESIMS, IR, UV, 1D/2D NMR, and CD spectra, are available in this publication and the Supplementary Materials.

## Supplementary Materials

The following supporting information can be downloaded at: https://www.mdpi.com/article/10.3390/molecules29030649/s1, Figure S1: HRESIMS spectrum of compound **1**; Figure S2: UV spectrum of compound **1**; Figure S3: ECD spectrum of compound **1**; Figure S4: IR spectrum of compound **1**; Figure S5: ^1^H NMR spectrum (500 MHz, CDCl_3_) of compound **1**; Figure S6: ^13^C NMR spectrum (125 MHz, CDCl_3_) of compound **1**; Figure S7: ^1^H-^1^H COSY spectrum of compound **1**; Figure S8: HSQC spectrum of compound **1**; Figure S9: HMBC spectrum of compound **1**; Figure S10: NOESY spectrum of compound **1**; Figure S11: ^1^H NMR spectrum (500 MHz, CD_3_OD) of compound **2**; Figure S12: ^13^C NMR spectrum (125 MHz, CD_3_OD) of compound **2**; Figure S13: ^1^H NMR spectrum (500 MHz, DMSO-*d*_6_) of compound **3**; Figure S14: ^13^C NMR spectrum (125 MHz, DMSO-*d*_6_) of compound **3**; Figure S15: ^1^H NMR spectrum (500 MHz, CDCl_3_) of compound **4**; Figure S16: ^13^C NMR spectrum (125 MHz, CDCl_3_) of compound **4**; Figure S17: ^1^H NMR spectrum (500 MHz, CDCl_3_) of compound **5**; Figure S18: ^13^C NMR spectrum (125 MHz, CDCl_3_) of compound **5**; Figure S19: ^1^H NMR spectrum (500 MHz, CDCl_3_) of compound **6**; Figure S20: ^13^C NMR spectrum (125 MHz, CDCl_3_) of compound **6**; Figure S21: ^1^H NMR spectrum (500 MHz, CDCl_3_) of compound **7**; Figure S22: ^13^C NMR spectrum (125 MHz, CDCl_3_) of compound **7**; Figure S23: ^1^H NMR spectrum (500 MHz, CDCl_3_) of compound **8**; Figure S24: ^13^C NMR spectrum (125 MHz, CDCl_3_) of compound **8**; Figure S25: ^1^H NMR spectrum (500 MHz, DMSO-*d*_6_) of compound **9**; Figure S26: ^13^C NMR spectrum (125 MHz, DMSO-*d*_6_) of compound **9**; Figure S27: ^1^H NMR spectrum (500 MHz, CD_3_OD) of compound **10**; Figure S28: ^13^C NMR spectrum (125 MHz, CD_3_OD) of compound **10**; Figure S29: ^1^H NMR spectrum (500 MHz, CDCl_3_) of compound **11**; Figure S30: ^13^C NMR spectrum (125 MHz, CDCl_3_) of compound **11**; Figure S31: ^1^H NMR spectrum (500 MHz, CDCl_3_) of compound **12**; Figure S32: ^13^C NMR spectrum (125 MHz, CDCl_3_) of compound **12**; Figure S33: ^1^H NMR spectrum (500 MHz, CDCl_3_) of compound **13**; Figure S34: ^13^C NMR spectrum (125 MHz, CDCl_3_) of compound **13**; Figure S35: ^1^H NMR spectrum (500 MHz, CDCl_3_) of compound **14**; Figure S36: ^13^C NMR spectrum (125 MHz, CDCl_3_) of compound **14**; Figure S37: ^1^H NMR spectrum (500 MHz, CDCl_3_) of compound **15**; Figure S38: ^13^C NMR spectrum (125 MHz, CDCl_3_) of compound **15**; Table S1: Evaluation of antibacterial activity of compounds **1**–**15**; Figure S39: 96-well plate antibacterial results; Table S2: Evaluation of antibacterial activity of compounds **4**; Excel: preliminary screening data of cytotoxic activity of compounds **1**–**15**.
