# Supplementary material for: Chemical Constituents and Bioactivities of the Plant-Derived Fungus Aspergillus fumigatus
Source: Molecules. 2024 Jan 30;29(3):649. doi: 10.3390/molecules29030649 (PMC10856792; doi:10.3390/molecules29030649)
Supplement: Supplementary file 1 [file molecules-29-00649-s001.zip › Supplementary Information1-29.pdf]

SUPPLEMENTARY MATERIAL

Chemical constituents and bioactivties of the Plant-Derived Fungus *Aspergillus fumigatus*

Zihuan Sang[1,2,3,†], Yangjiang Zhang[3,†], Kaidi Qiu[3], Yuting Zheng[1], Chen Chen[1,2,3], Li Xu[1], Jiaying Lai [3], Zhenxing Zou[1,*], Haibo Tan[1,2,3,*]

[1] Hunan Key Laboratory of Diagnostic and Therapeutic Drug Rsearch for Chronic Diseases, Xiangya School of Pharmaceutical Sciences, Central South University, Changsha 410013, China

[2] National Engineering Research Center of Navel Orange, Gannan Normal University, Ganzhou 341000, People's Republic of China

[3] Key Laboratory of South China Agricultural Plant Molecular Analysis and Genetic Improvement, Guangdong Provincial Key Laboratory of Applied Botany, South China Botanical Garden, Chinese Academy of Sciences, Guangzhou 510650, China

* Correspondence: zouzhenxing@csu.edu.cn (Z.Z.); tanhaibo@scbg.ac.cn (H.T.);
Tel.: +86-183-1851-7352 (H.T.)

† These authors contributed equally to this work.

Contents

Figure S1. HRESIMS spectrum of compound **1**.

Figure S2. UV spectrum of compound **1**.

Figure S3. ECD spectrum of compound **1**.

Figure S4. IR spectrum of compound **1**.

Figure S5. ^{1}H NMR spectrum (500 MHz, $CDCl_3$) of compound **1**.

Figure S6. ^{13}C NMR spectrum (125 MHz, $CDCl_3$) of compound **1**.

Figure S7. ^{1}H-^{1}H COSY spectrum of compound **1**.

Figure S8. HSQC spectrum of compound **1**.

Figure S9. HMBC spectrum of compound **1**.

Figure S10. NOESY spectrum of compound **1**.

Figure S11. ^{1}H NMR spectrum (500 MHz, CD_3OD:$CDCl_3$-1:1) of compound **2**.

Figure S12. ^{13}C NMR spectrum (125 MHz, CD_3OD:$CDCl_3$-1:1) of compound **2**.

Figure S13. ^{1}H NMR spectrum (500 MHz, DMSO-d_6) of compound **3**.

Figure S14. ^{13}C NMR spectrum (125 MHz, DMSO-d_6) of compound **3**.

Figure S15. ^{1}H NMR spectrum (500 MHz, $CDCl_3$) of compound **4**.

Figure S16. ^{13}C NMR spectrum (125 MHz, $CDCl_3$) of compound **4**.

Figure S17. ^{1}H NMR spectrum (500 MHz, $CDCl_3$) of compound **5**.

Figure S18. ^{13}C NMR spectrum (125 MHz, $CDCl_3$) of compound **5**.

Figure S19. ^{1}H NMR spectrum (500 MHz, $CDCl_3$) of compound **6**.

Figure S20. ^{13}C NMR spectrum (125 MHz, $CDCl_3$) of compound **6**.

Figure S21. ^{1}H NMR spectrum (500 MHz, $CDCl_3$) of compound **7**.

Figure S22. ^{13}C NMR spectrum (125 MHz, $CDCl_3$) of compound **7**.

Figure S23. ^{1}H NMR spectrum (500 MHz, $CDCl_3$) of compound **8**.

Figure S24. ^{13}C NMR spectrum (125 MHz, $CDCl_3$) of compound **8**.

Figure S25. ^{1}H NMR spectrum (500 MHz, DMSO-d_6) of compound **9**.

Figure S26. ^{13}C NMR spectrum (125 MHz, DMSO-d_6) of compound **9**.

Figure S27. ^{1}H NMR spectrum (500 MHz, CD_3OD) of compound **10**.

Figure S28. ^{13}C NMR spectrum (125 MHz, CD_3OD) of compound **10**.

Figure S29. ^{1}H NMR spectrum (500 MHz, $CDCl_3$) of compound **11**.

Figure S30. ^{13}C NMR spectrum (125 MHz, $CDCl_3$) of compound **11**.

Figure S31. ^{1}H NMR spectrum (500 MHz, $CDCl_3$) of compound **12**.

Figure S32. ^{13}C NMR spectrum (125 MHz, $CDCl_3$) of compound **12**.

Figure S33. ^{1}H NMR spectrum (500 MHz, $CDCl_3$) of compound **13**.

Figure S34. ^{13}C NMR spectrum (125 MHz, $CDCl_3$) of compound **13**.

Figure S35. ^{1}H NMR spectrum (500 MHz, $CDCl_3$) of compound **14**.

Figure S36. ^{13}C NMR spectrum (125 MHz, $CDCl_3$) of compound **14**.

Figure S37. ^{1}H NMR spectrum (500 MHz, $CDCl_3$) of compound **15**.

Figure S38. ^{13}C NMR spectrum (125 MHz, $CDCl_3$) of compound **15**.

Table S1. Evaluation of antibacterial activity of compounds **1-15**.

Figure S39. 96-well plate antibacterial results.

Table S2. Evaluation of antibacterial activity of compounds **4**.

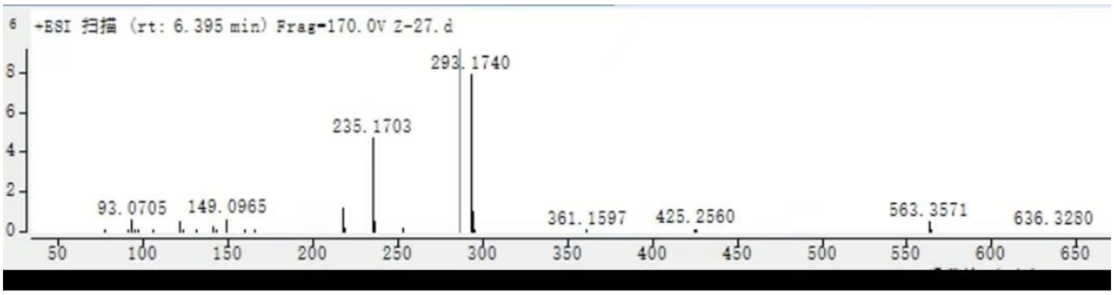

Figure S1. HRESIMS spectrum of compound **1**.

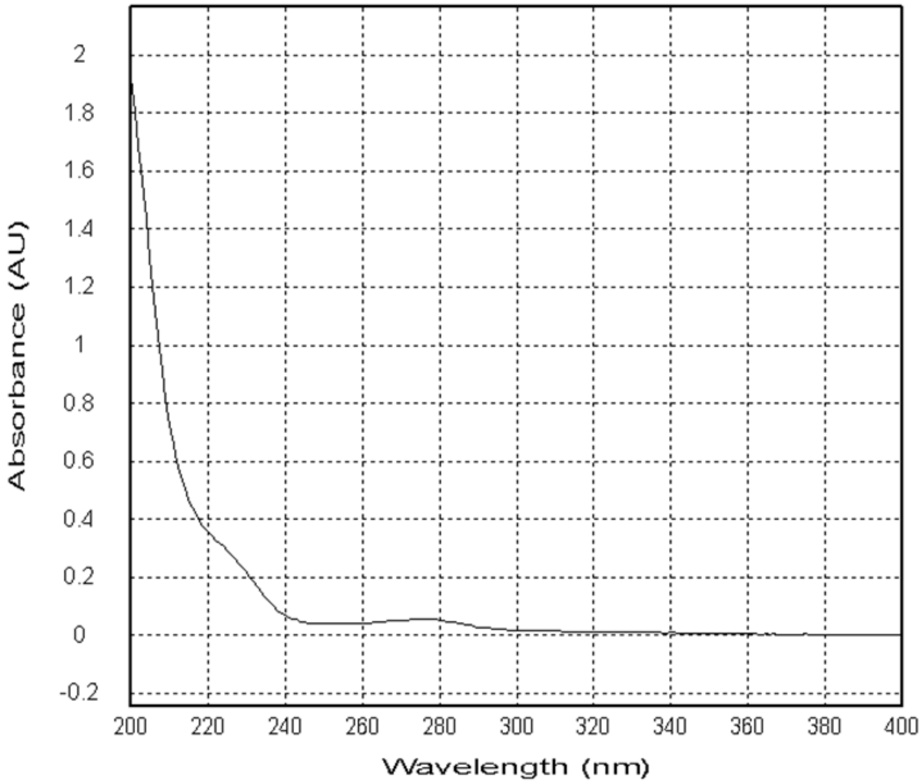

Figure S2. UV spectrum of compound **1**.

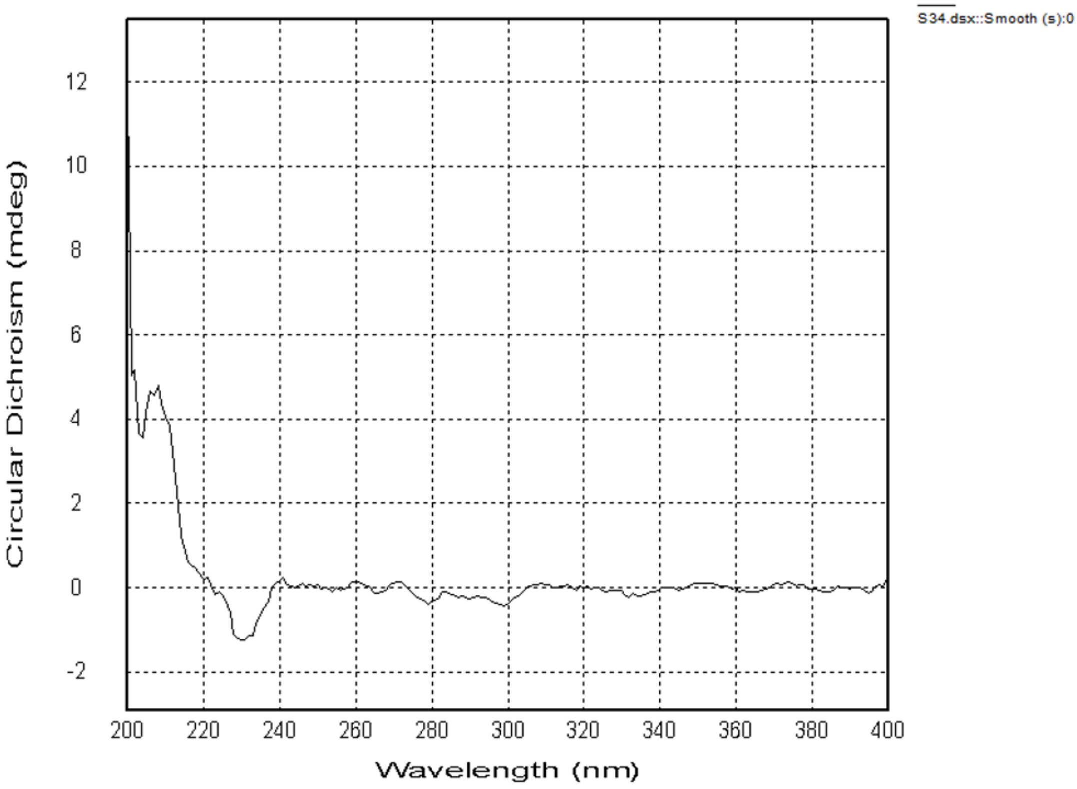

Figure S3. ECD spectrum of compound **1**.

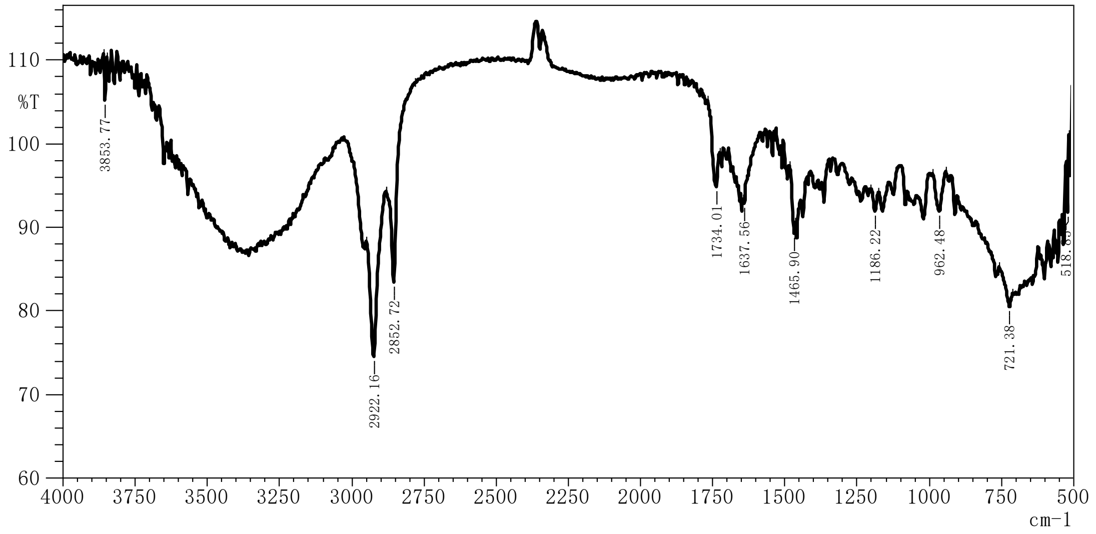

Figure S4. IR spectrum of compound **1**.

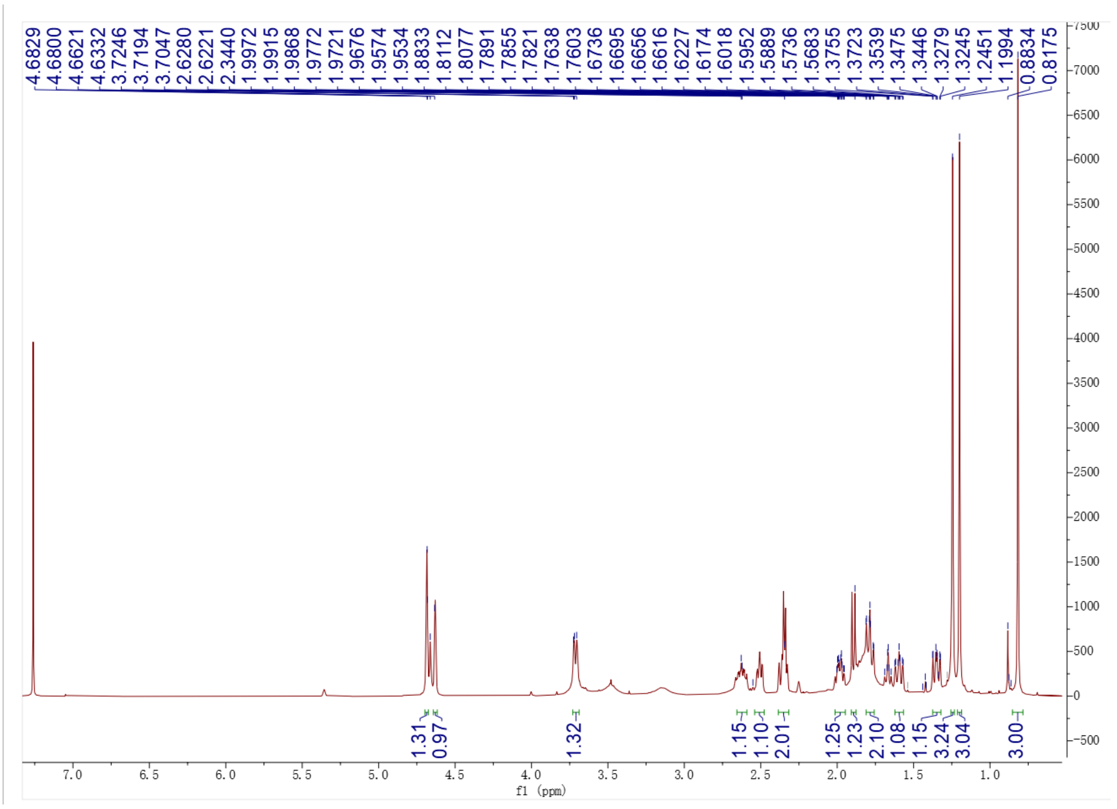

Figure S5. ^{1}H NMR spectrum (500 MHz, $CDCl_3$) of compound **1**.

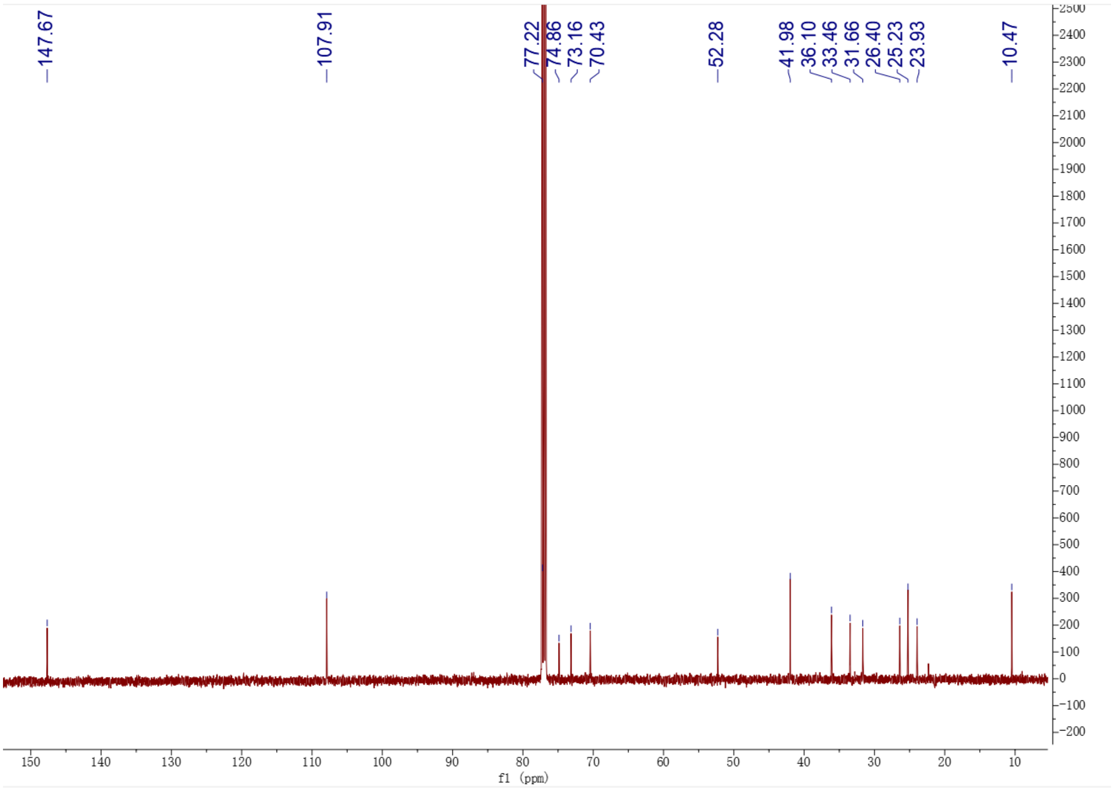

Figure S6. ^{13}C NMR spectrum (125 MHz, $CDCl_3$) of compound **1**.

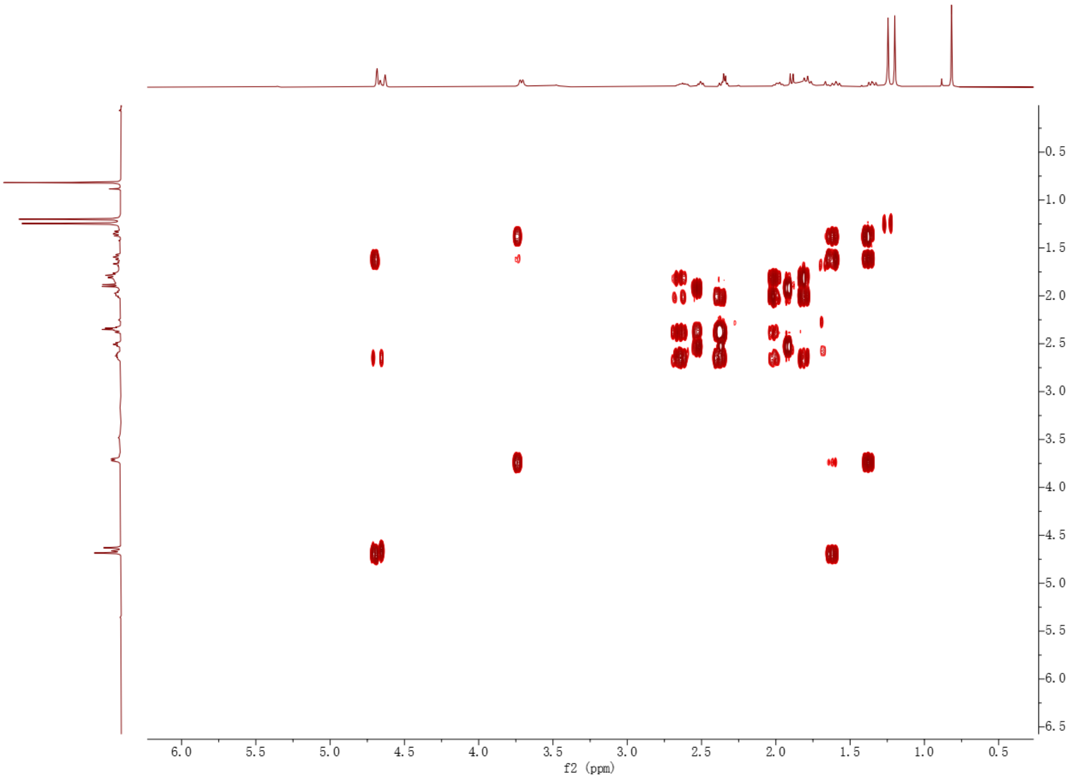

Figure S7. ^{1}H-^{1}H COSY spectrum of compound **1**.

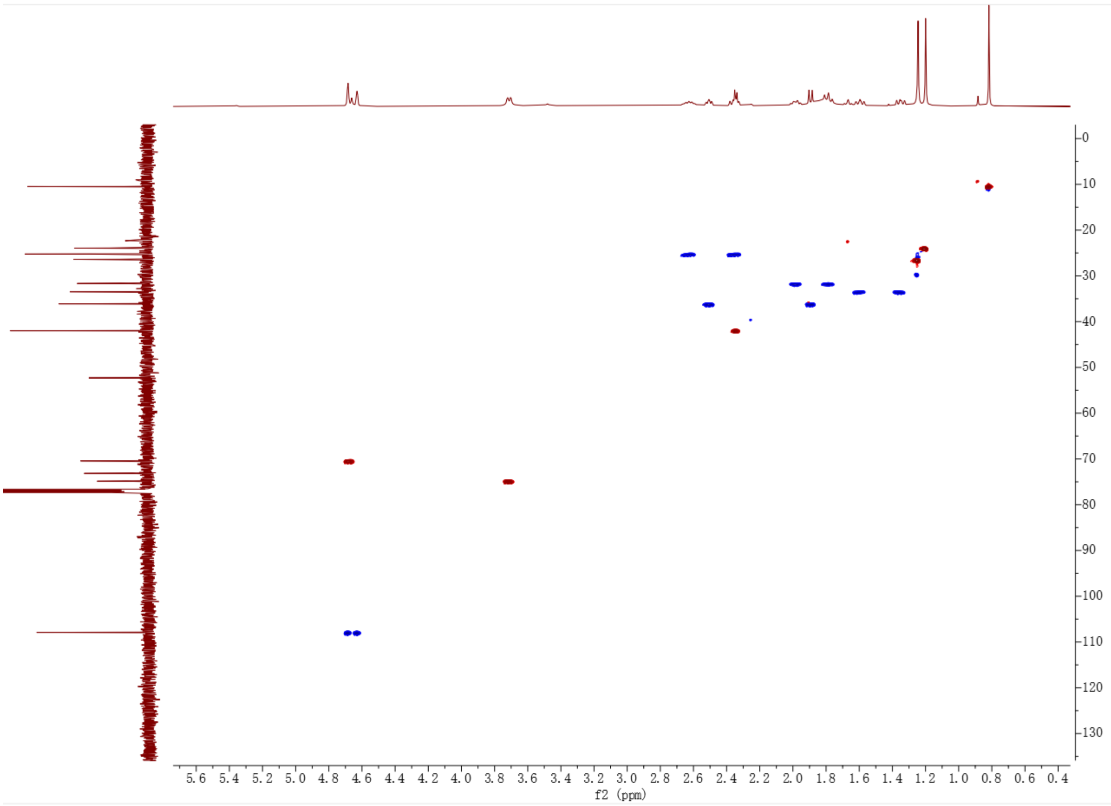

Figure S8. HSQC spectrum of compound **1**.

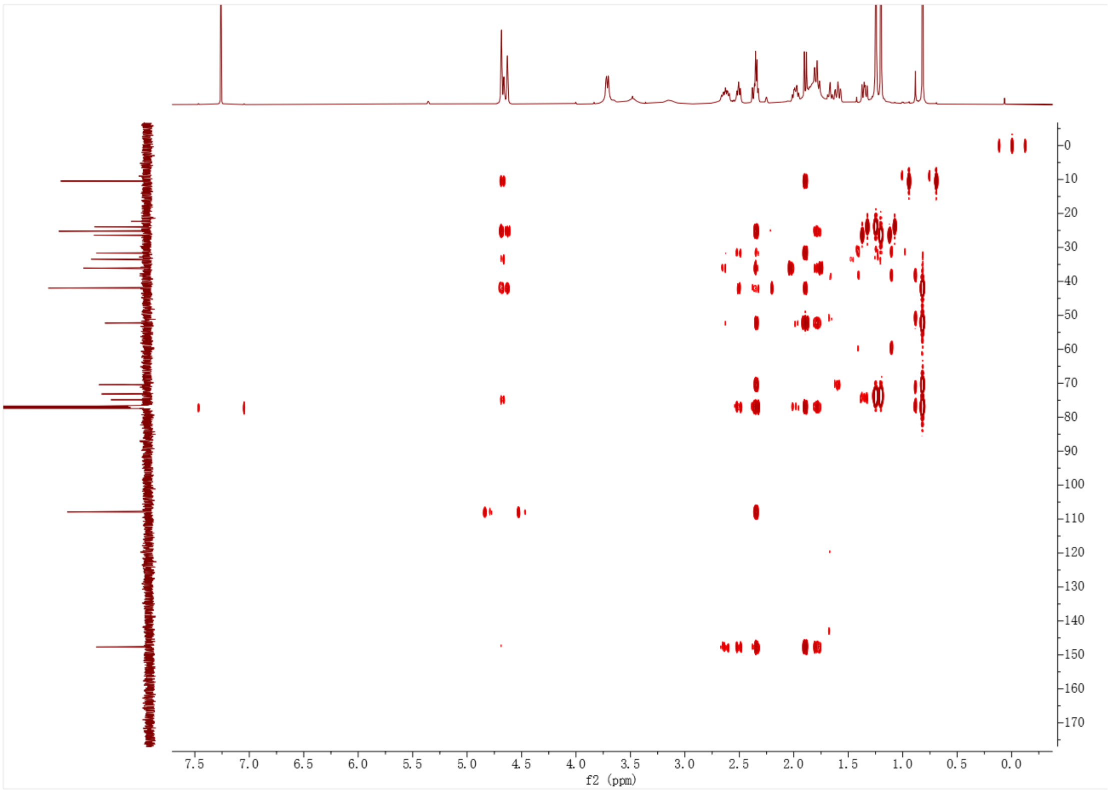

Figure S9. HMBC spectrum of compound **1**.

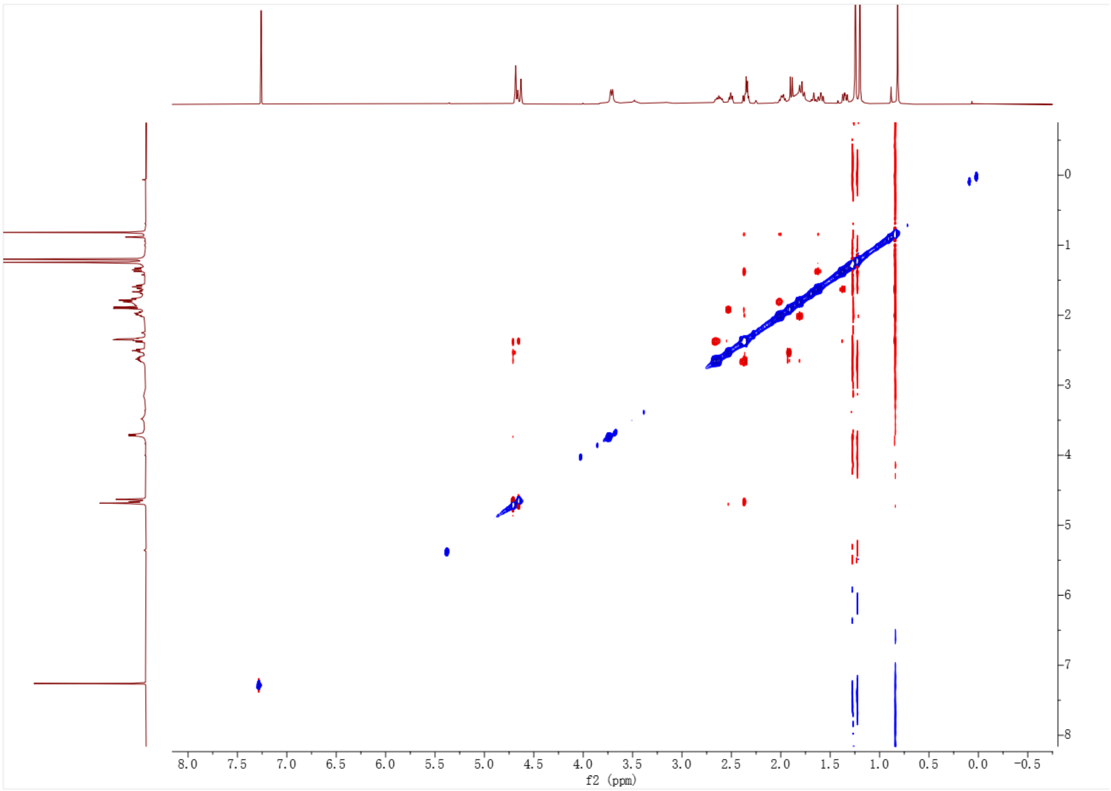

Figure S10. NOESY spectrum of compound **1**.

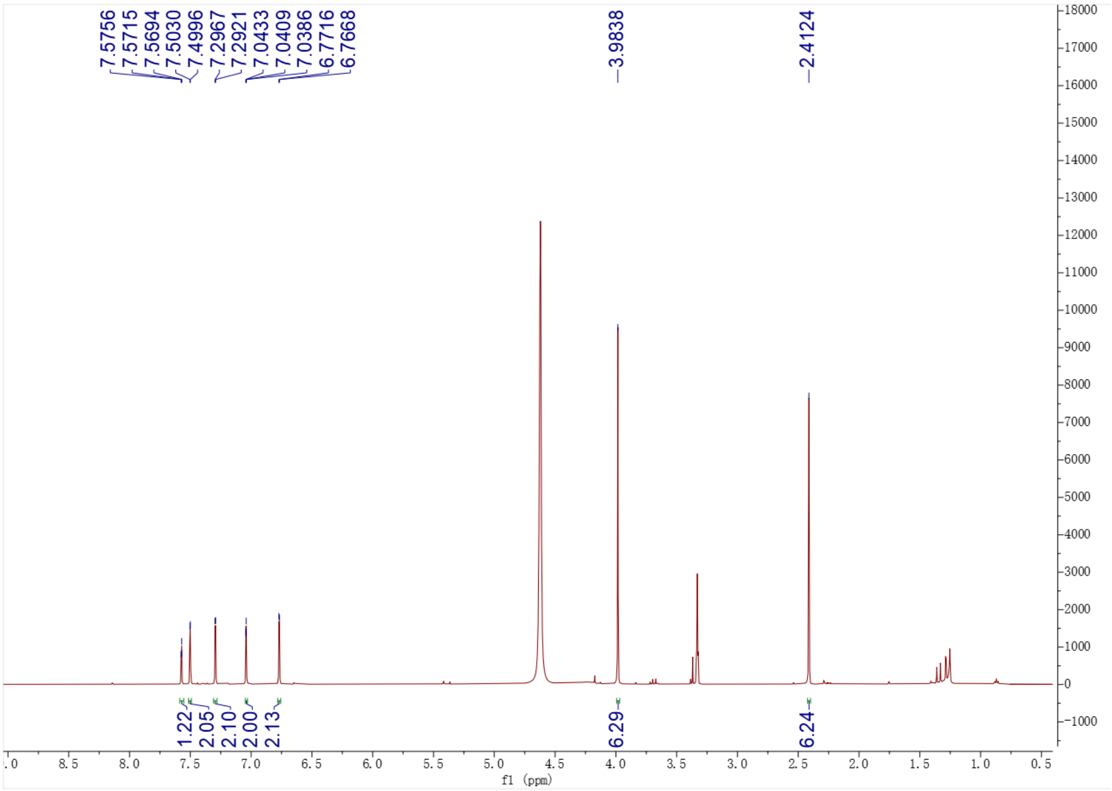

Figure S11. ¹H NMR spectrum (500 MHz, $CD_3OD:CDCl_3$-1:1) of compound **2**.

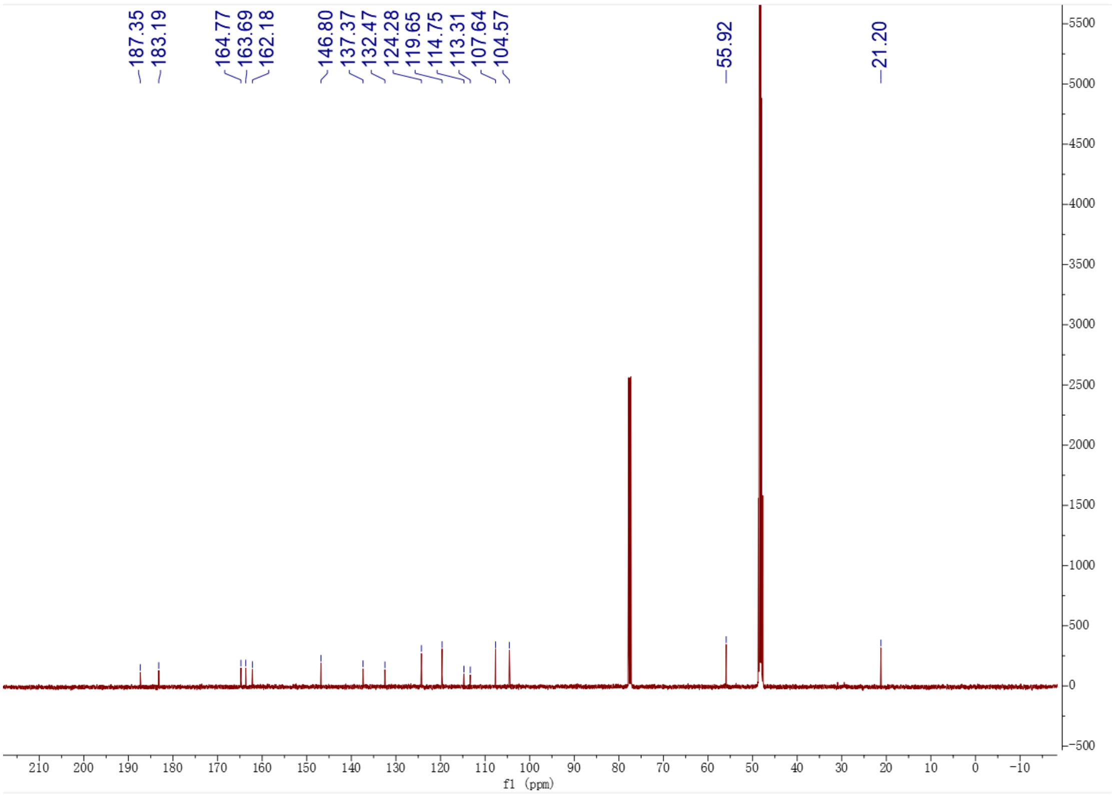

Figure S12. ¹³C NMR spectrum (125 MHz, $CD_3OD:CDCl_3$-1:1) of compound **2**.

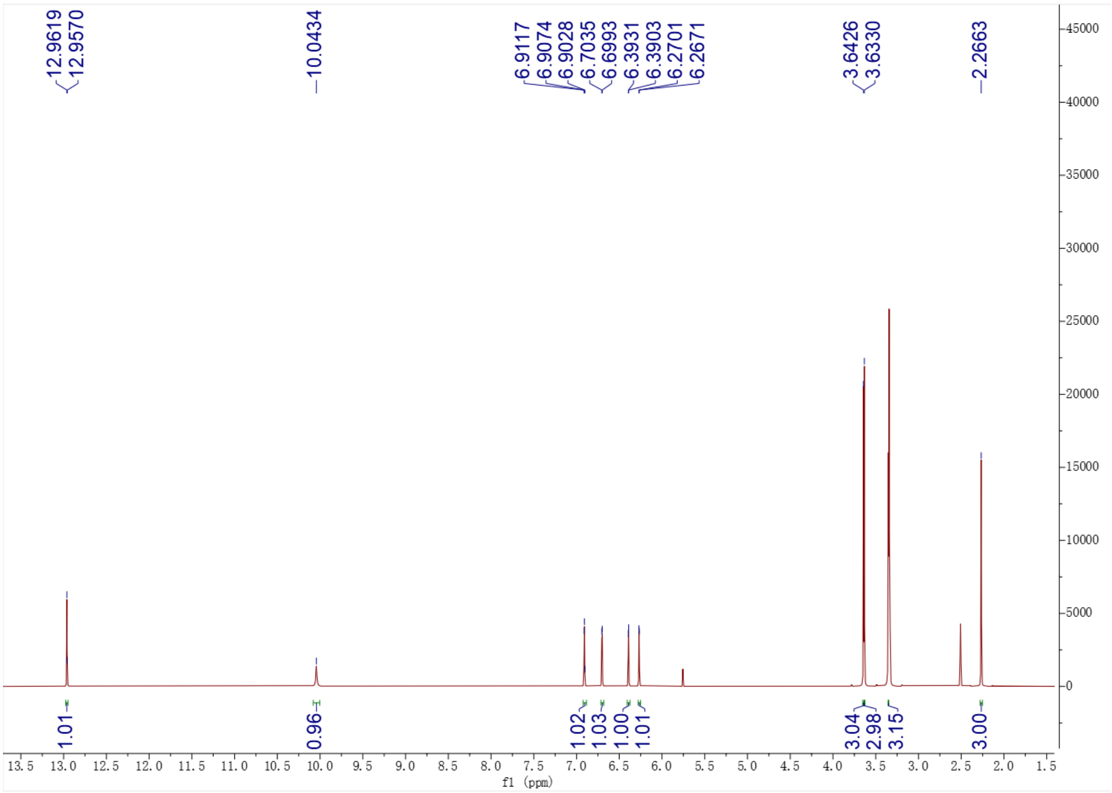

Figure S13. ^{1}H NMR spectrum (500 MHz, DMSO-d_6) of compound **3**.

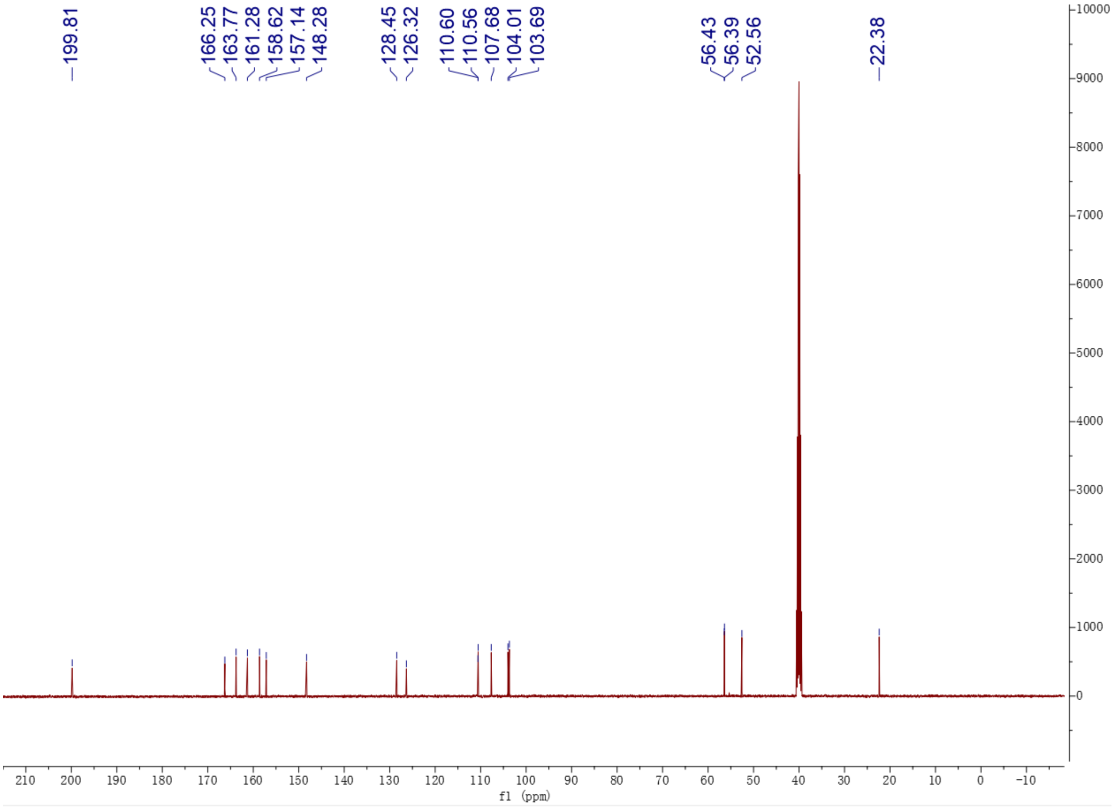

Figure S14. ^{13}C NMR spectrum (125 MHz, DMSO-d_6) of compound **3**.

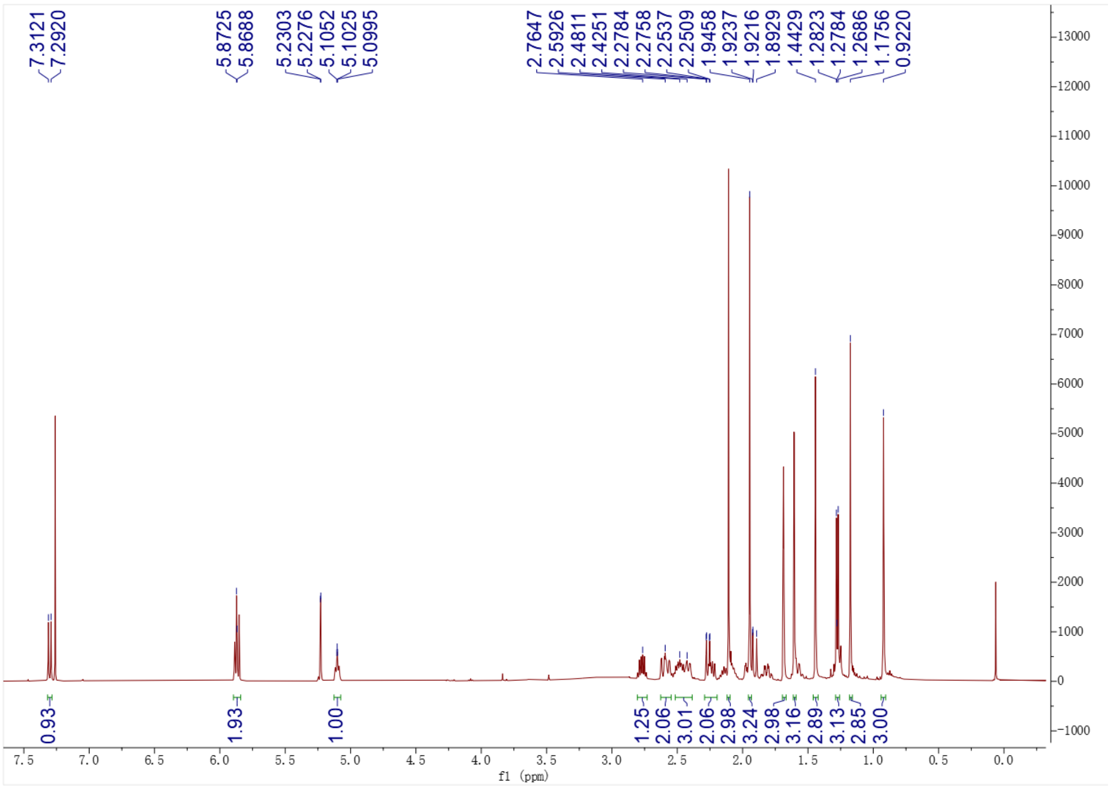

Figure S15. ^{1}H NMR spectrum (500 MHz, $CDCl_3$) of compound **4**.

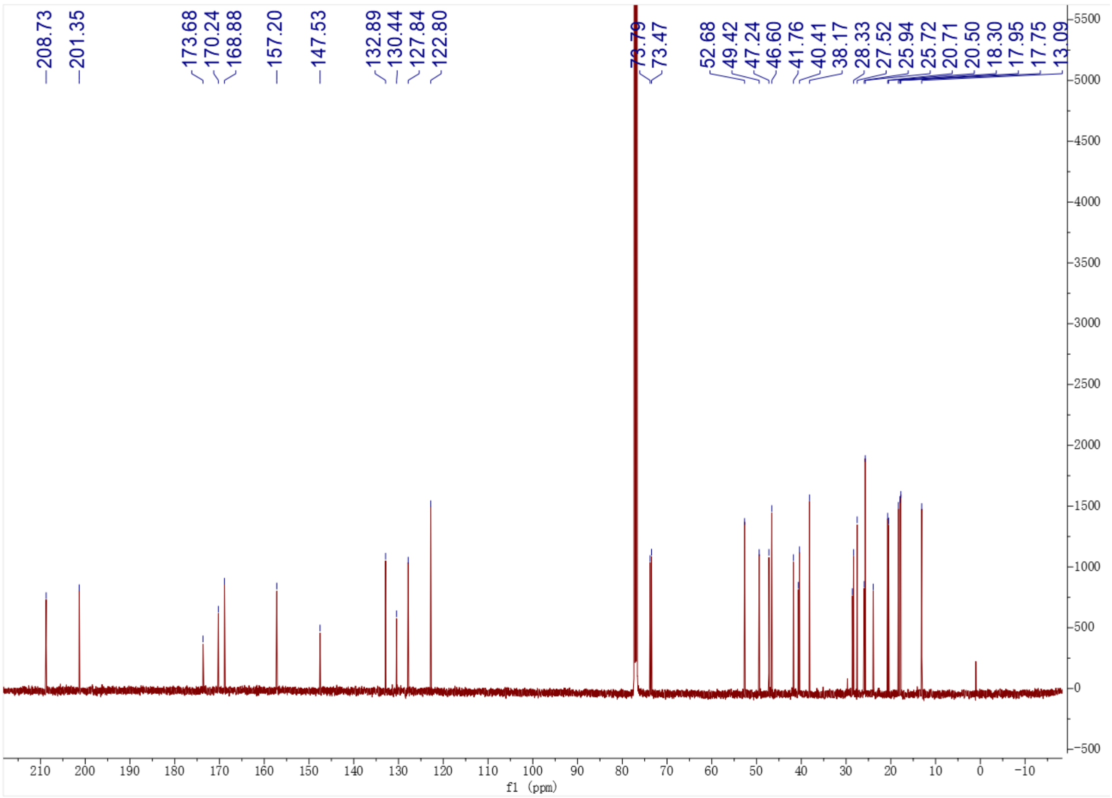

Figure S16. ^{13}C NMR spectrum (125 MHz, $CDCl_3$) of compound **4**.

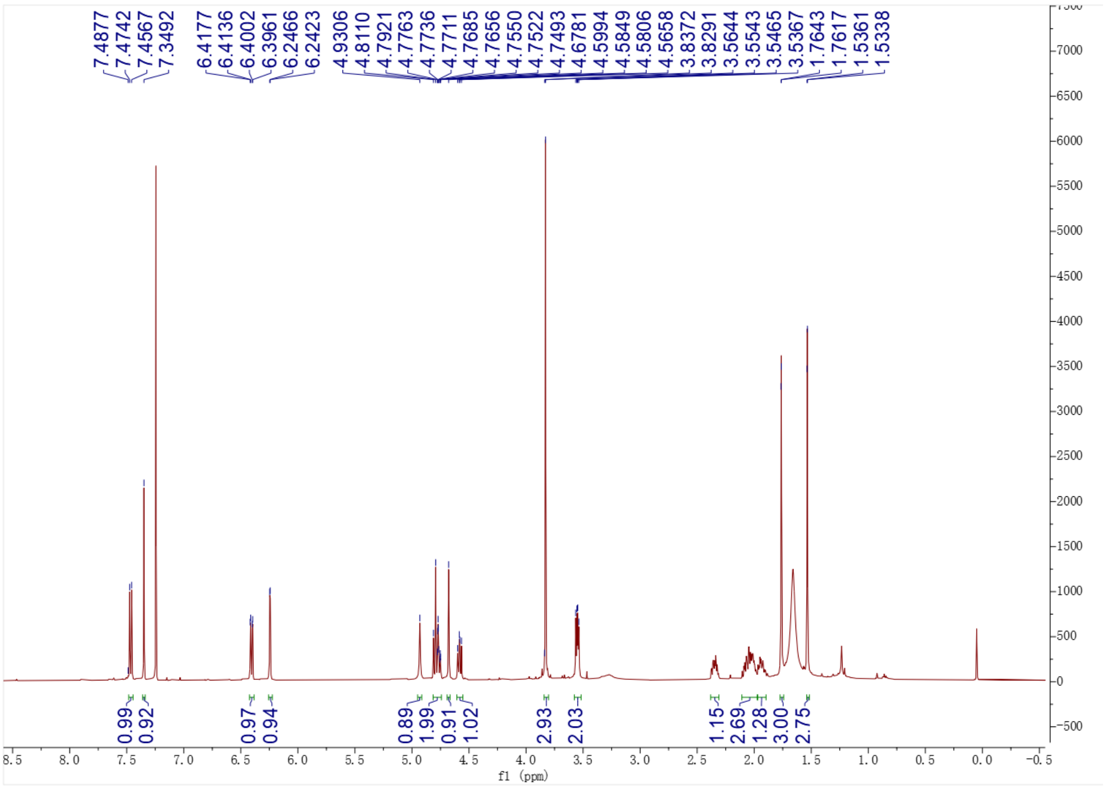

Figure S17. ^{1}H NMR spectrum (500 MHz, $CDCl_3$) of compound **5**.

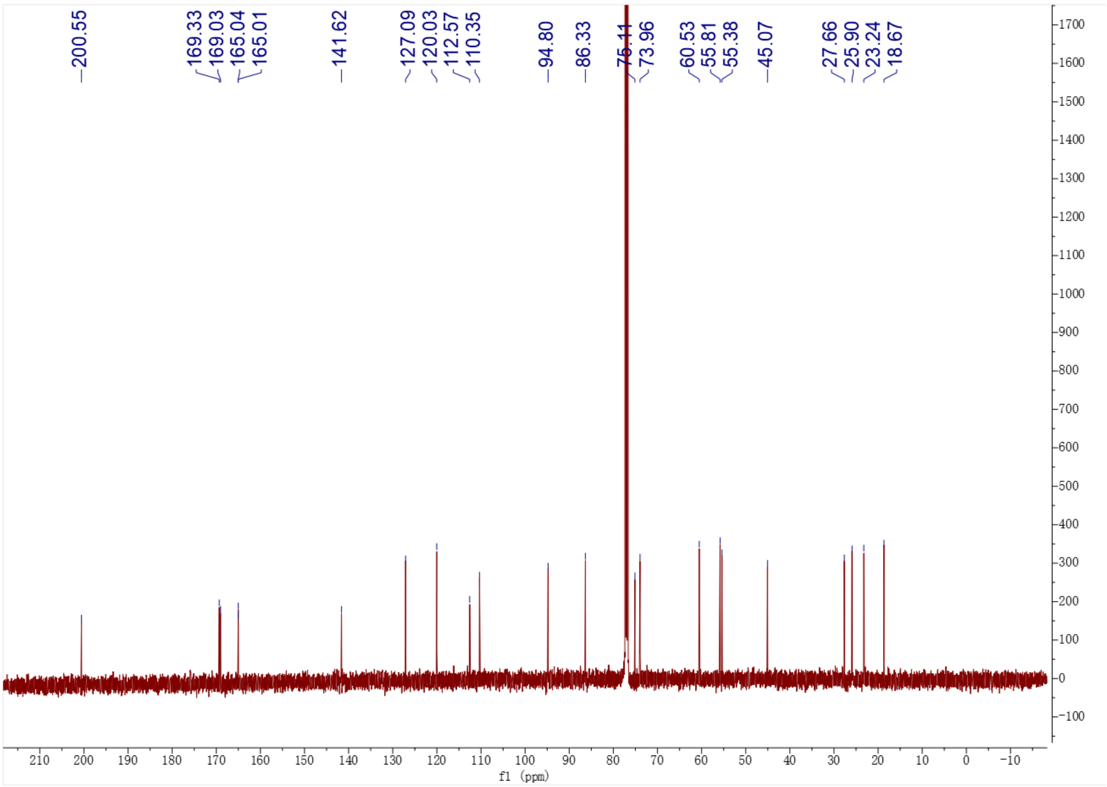

Figure S18. ^{13}C NMR spectrum (125 MHz, $CDCl_3$) of compound **5**.

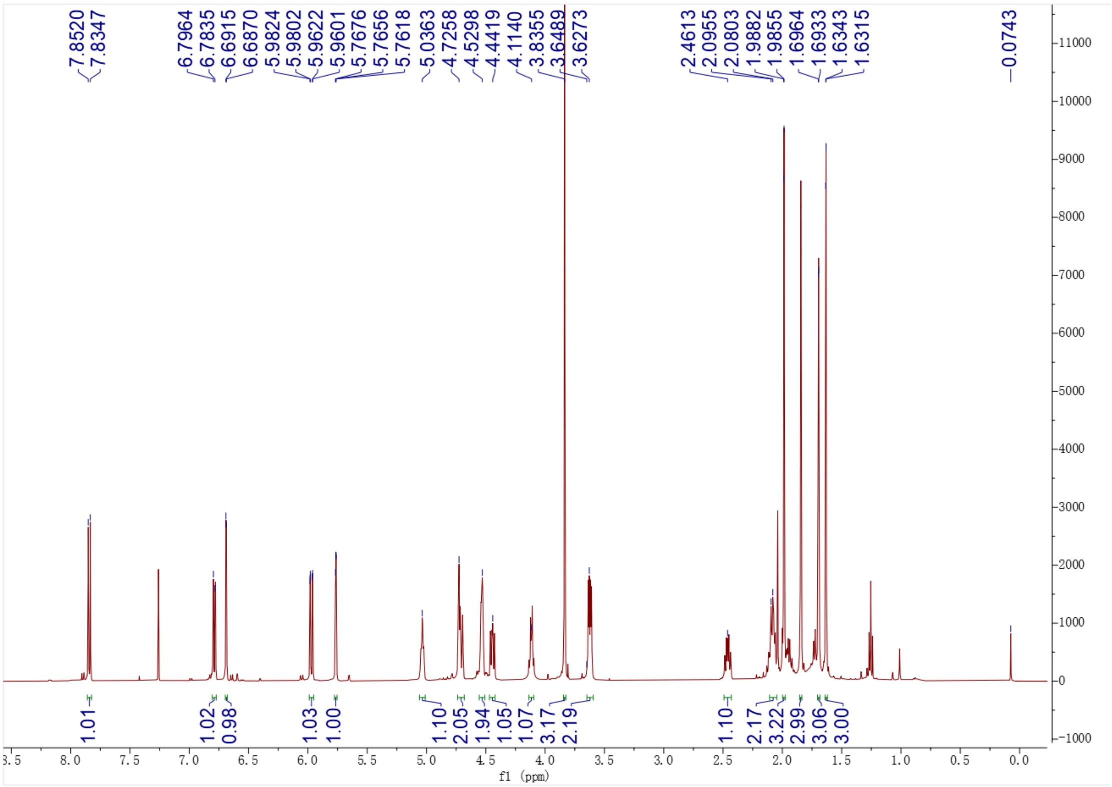

Figure S19. ^{1}H NMR spectrum (500 MHz, $CDCl_3$) of compound **6**.

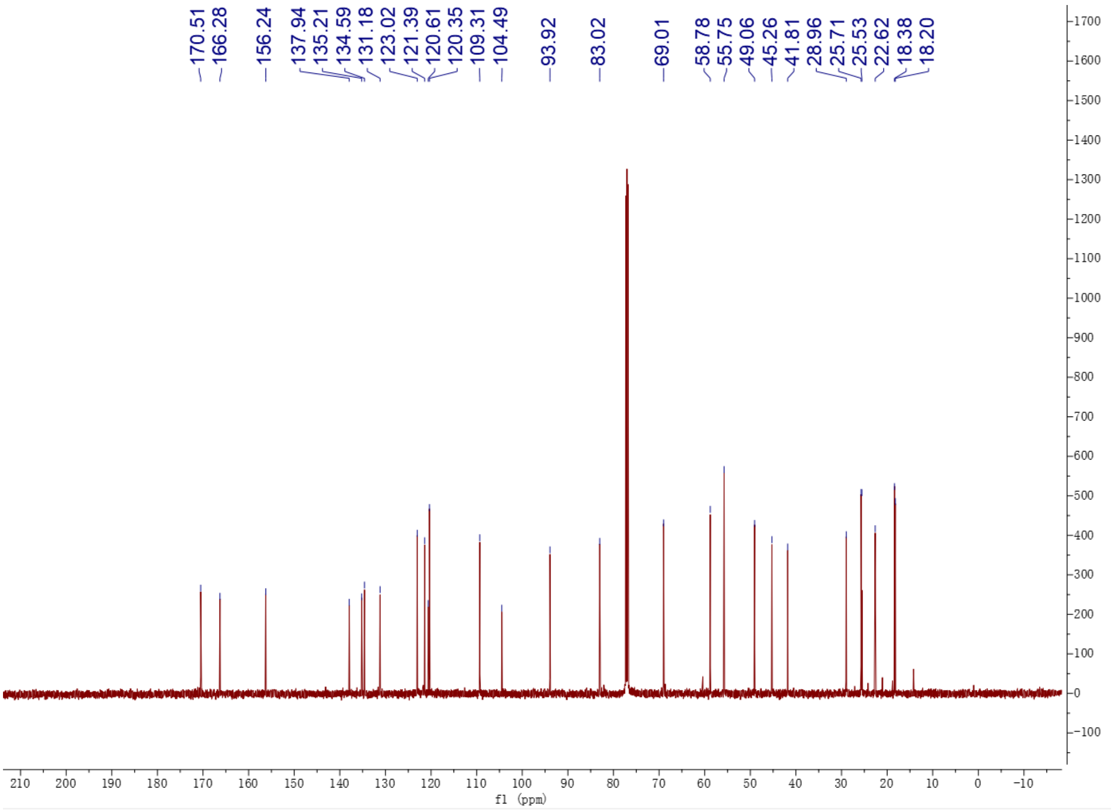

Figure S20. ^{13}C NMR spectrum (125 MHz, $CDCl_3$) of compound **6**.

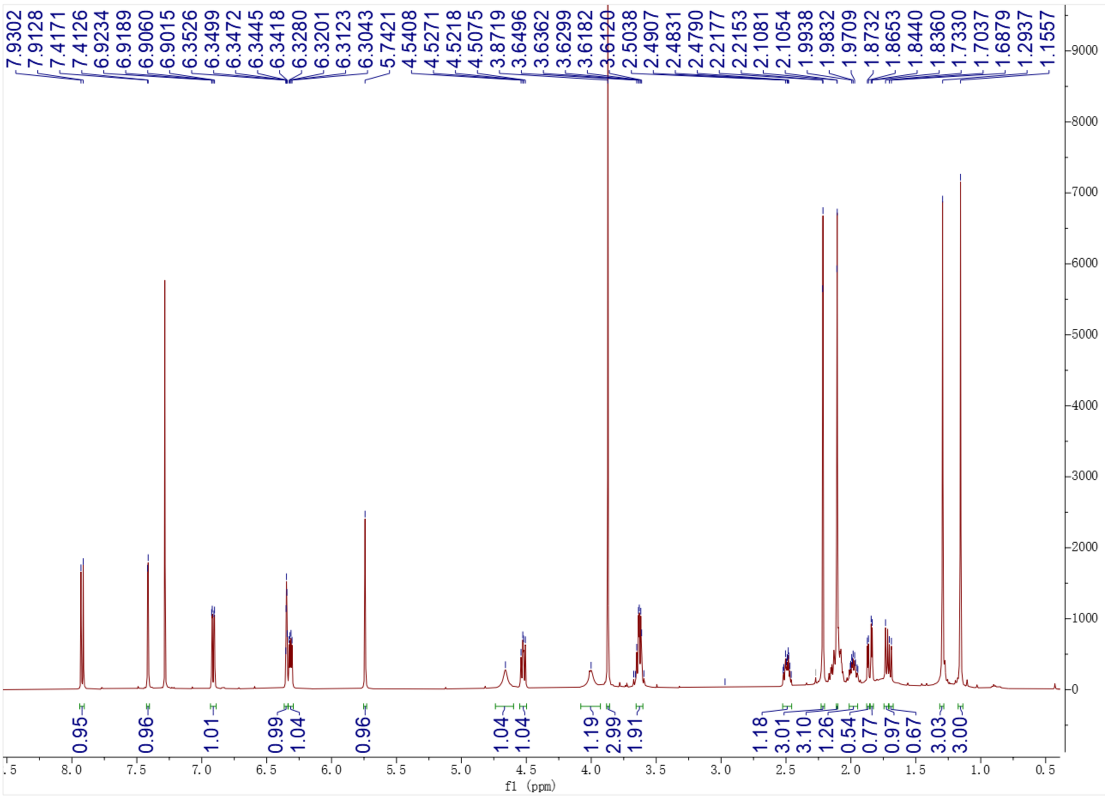

Figure S21. ^{1}H NMR spectrum (500 MHz, $CDCl_3$) of compound **7**.

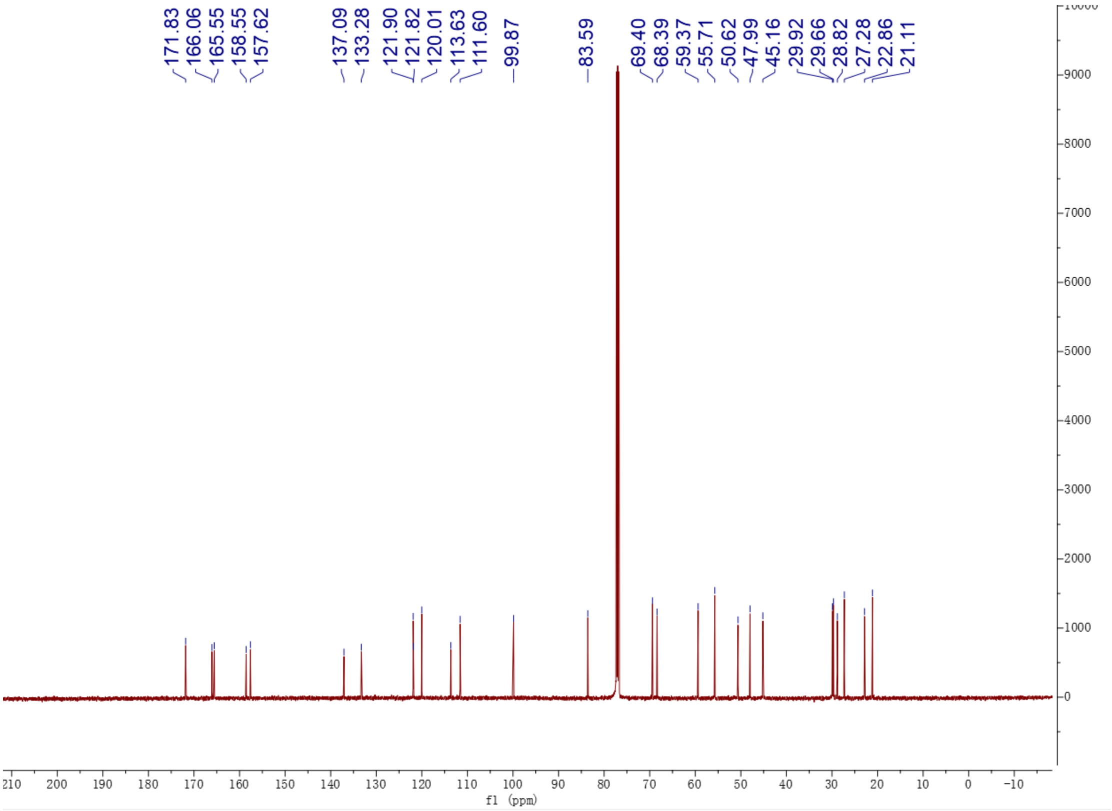

Figure S22. ^{13}C NMR spectrum (125 MHz, $CDCl_3$) of compound **7**.

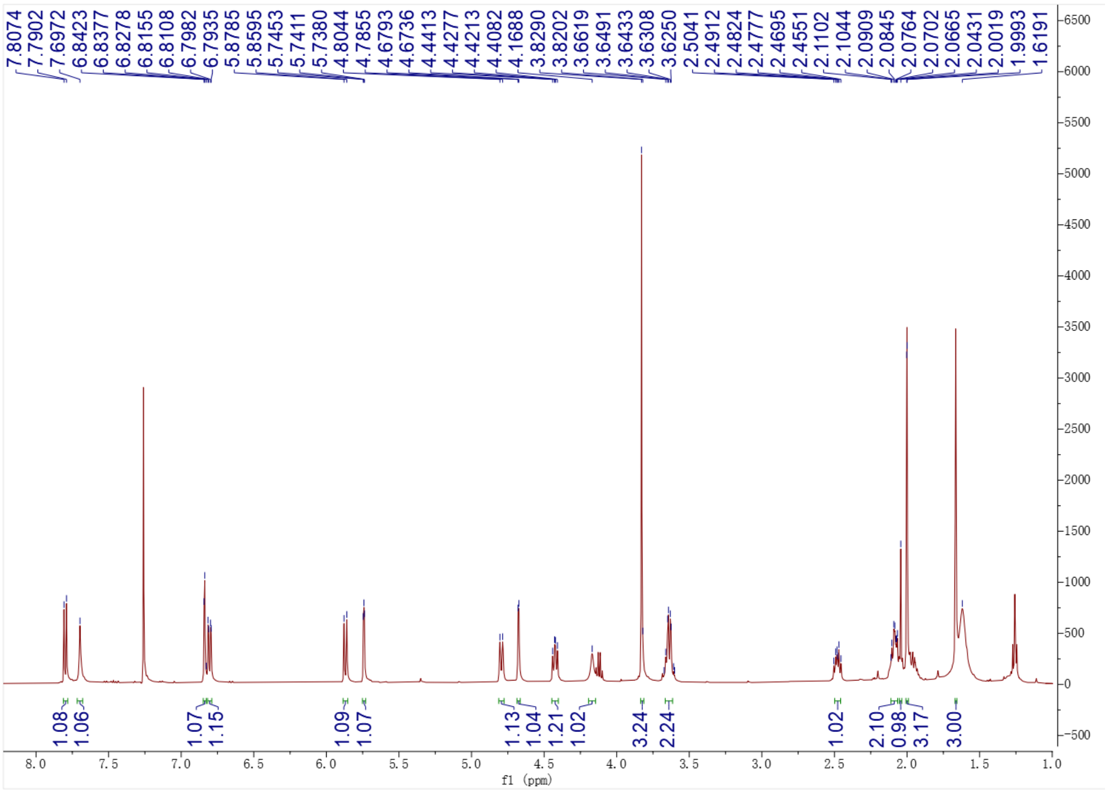

Figure S23. ^{1}H NMR spectrum (500 MHz, $CDCl_3$) of compound **8**.

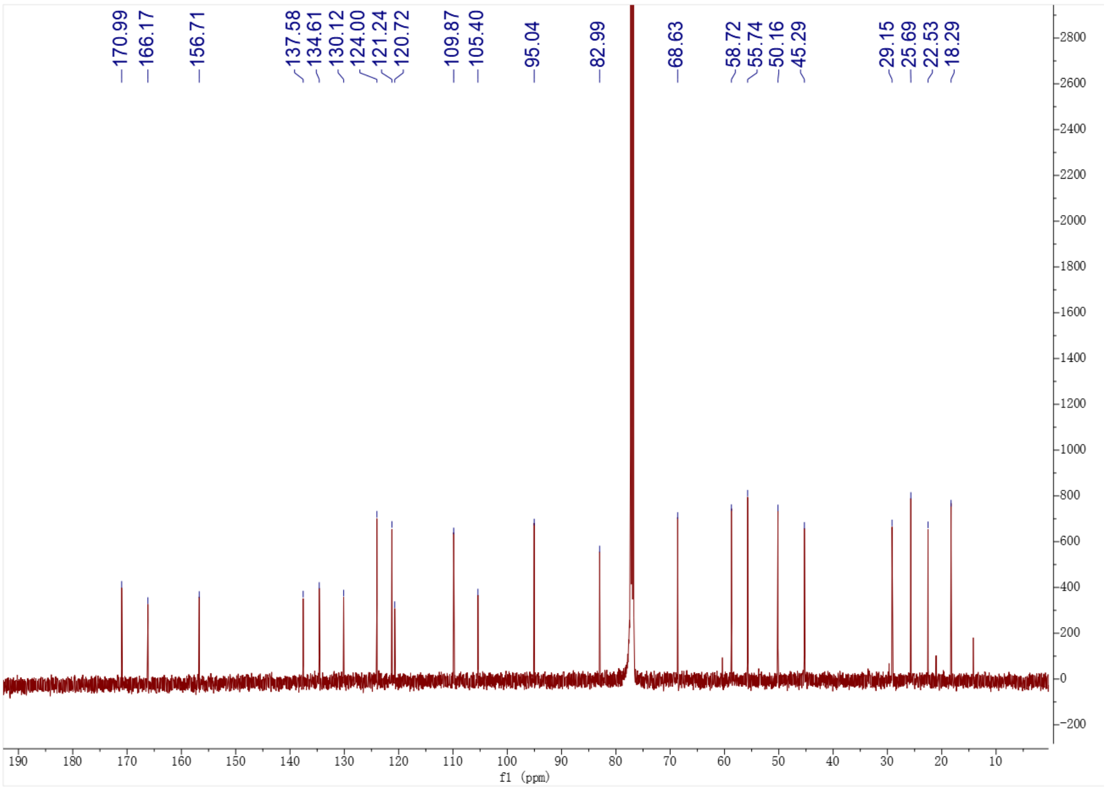

Figure S24. ^{13}C NMR spectrum (125 MHz, $CDCl_3$) of compound **8**.

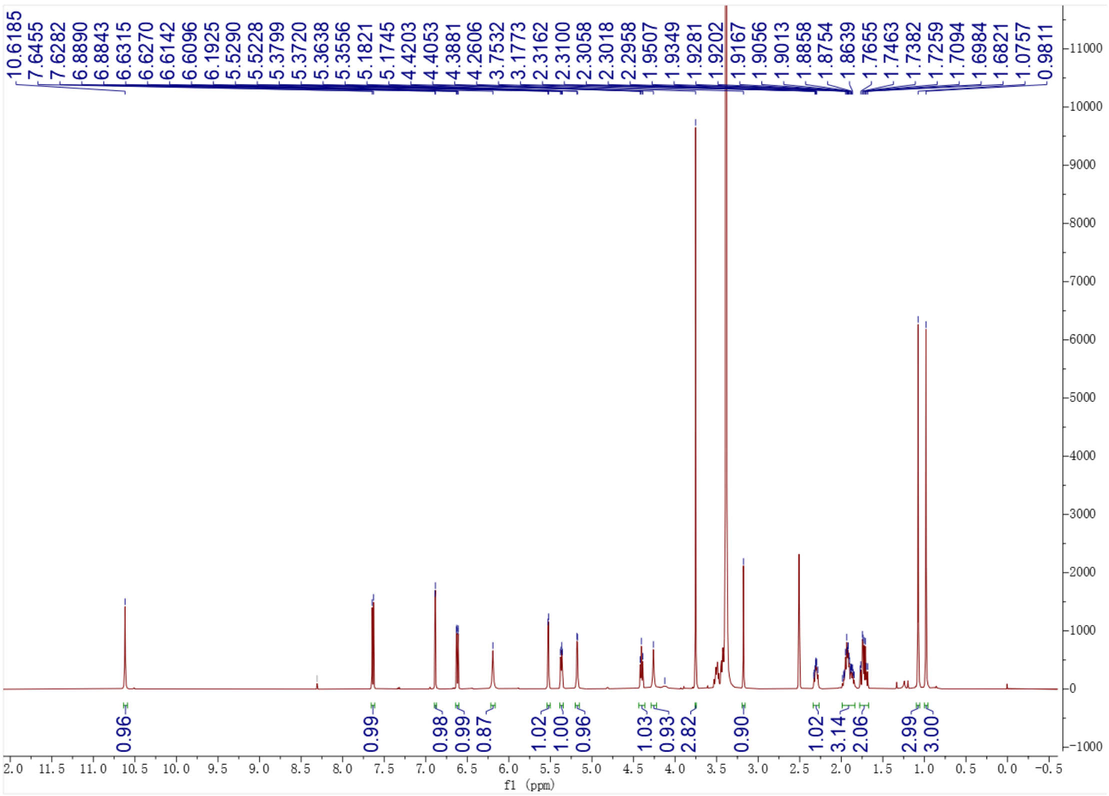

Figure S25. ^{1}H NMR spectrum (500 MHz, DMSO-d_6) of compound **9**.

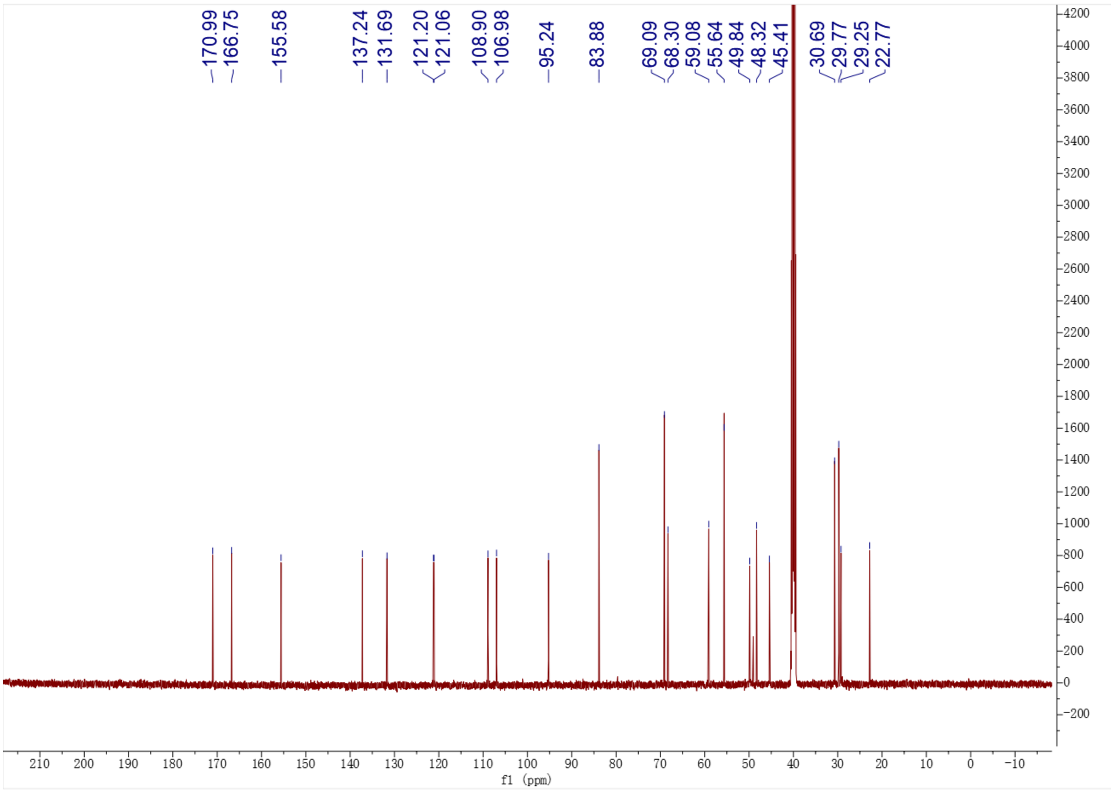

Figure S26. ^{13}C NMR spectrum (125 MHz, DMSO-d_6) of compound **9**.

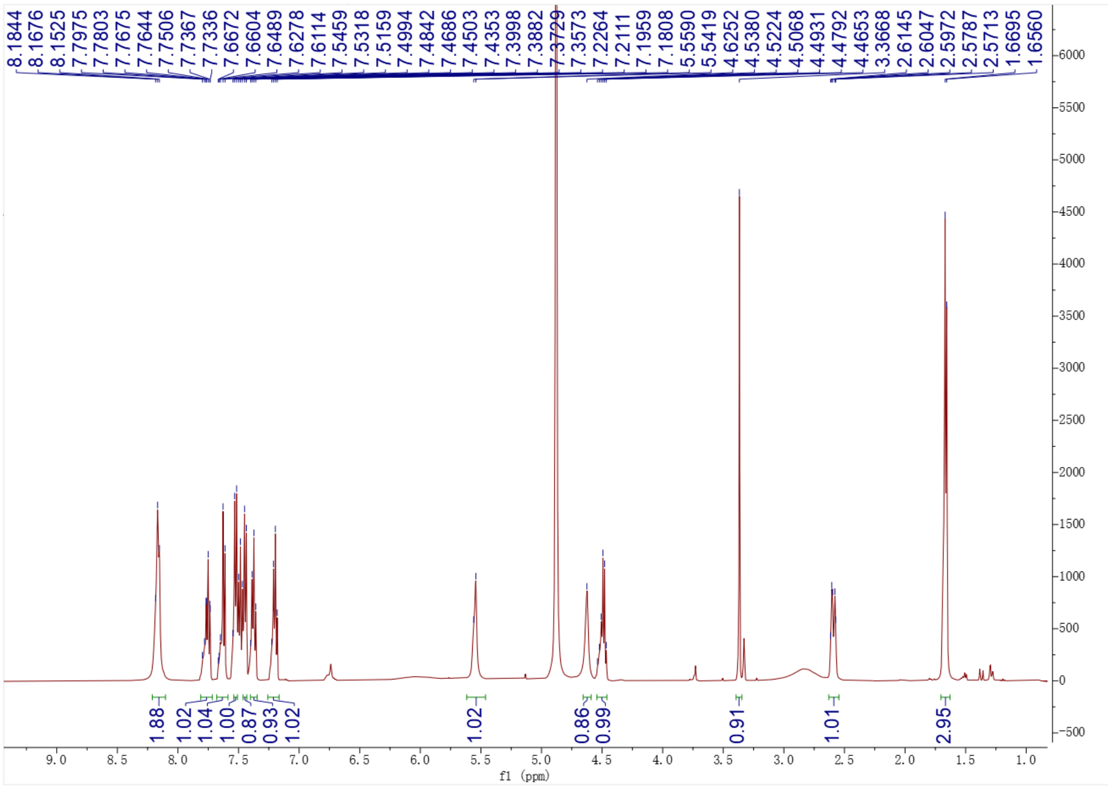

Figure S27. ^{1}H NMR spectrum (500 MHz, CD_3OD) of compound **10**.

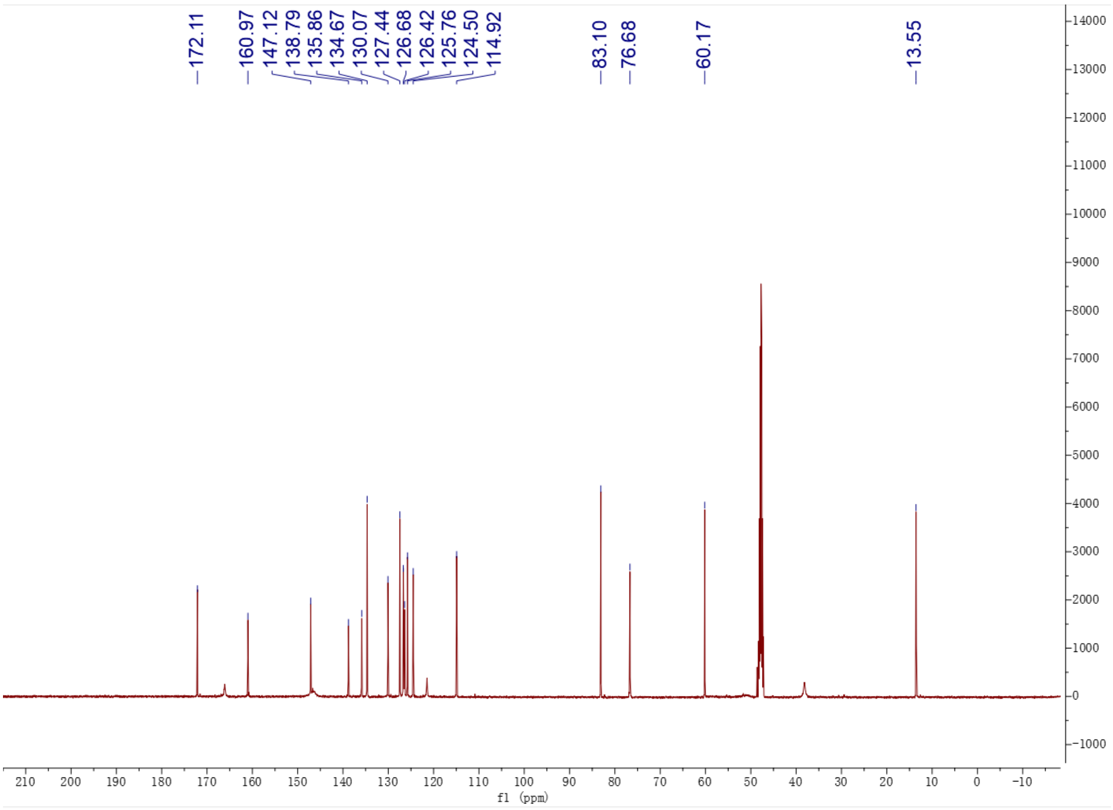

Figure S28. ^{13}C NMR spectrum (125 MHz, CD_3OD) of compound **10**.

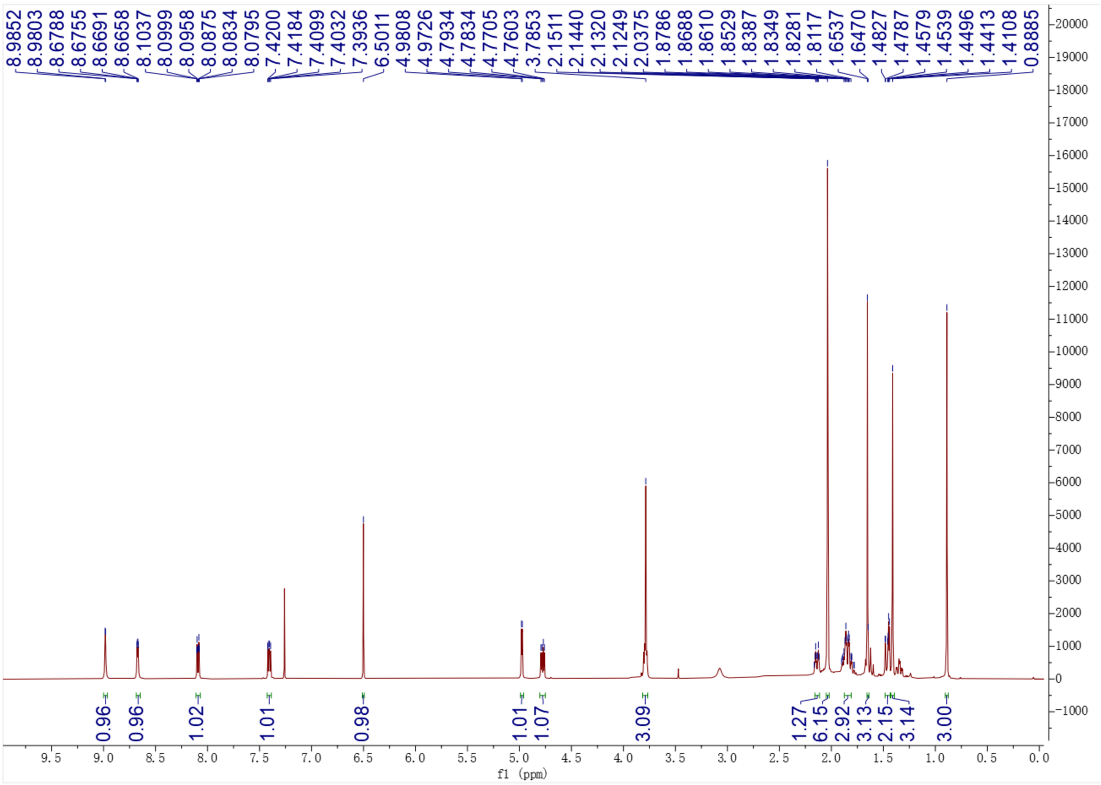

Figure S29. ^{1}H NMR spectrum (500 MHz, $CDCl_3$) of compound **11**.

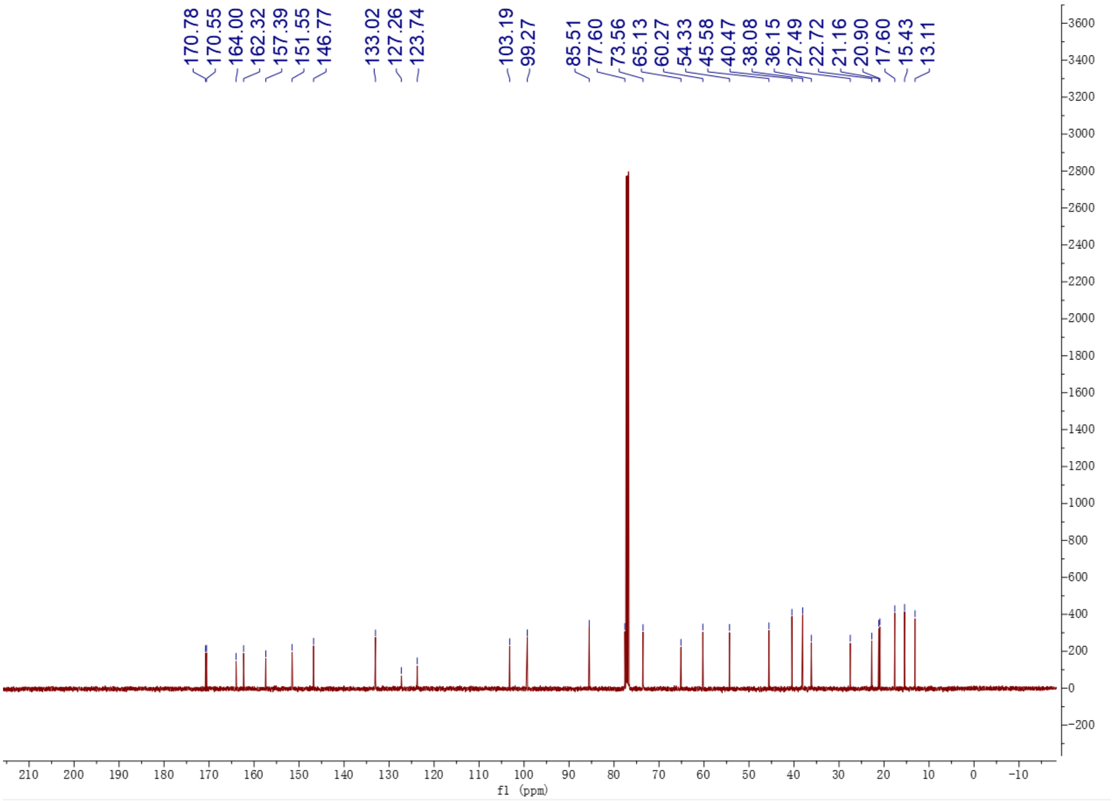

Figure S30. ^{13}C NMR spectrum (125 MHz, $CDCl_3$) of compound **11**.

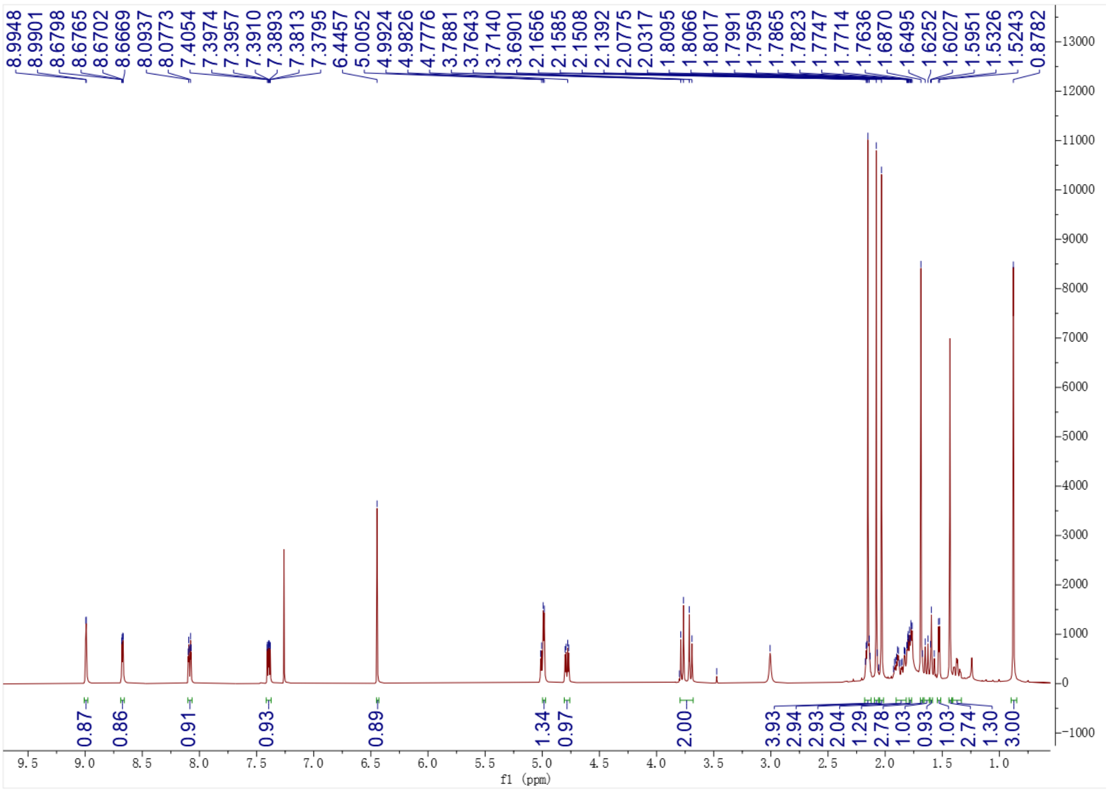

Figure S31. ^{1}H NMR spectrum (500 MHz, $CDCl_3$) of compound **12**.

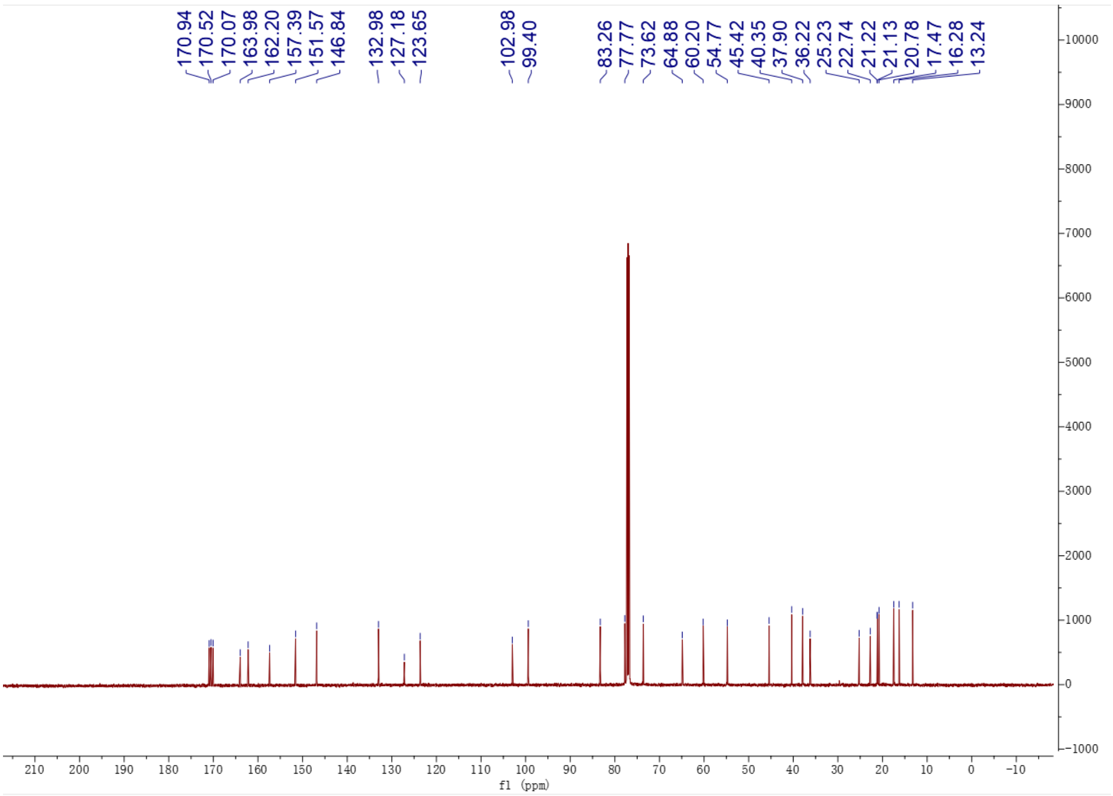

Figure S32. ^{13}C NMR spectrum (125 MHz, $CDCl_3$) of compound **12**.

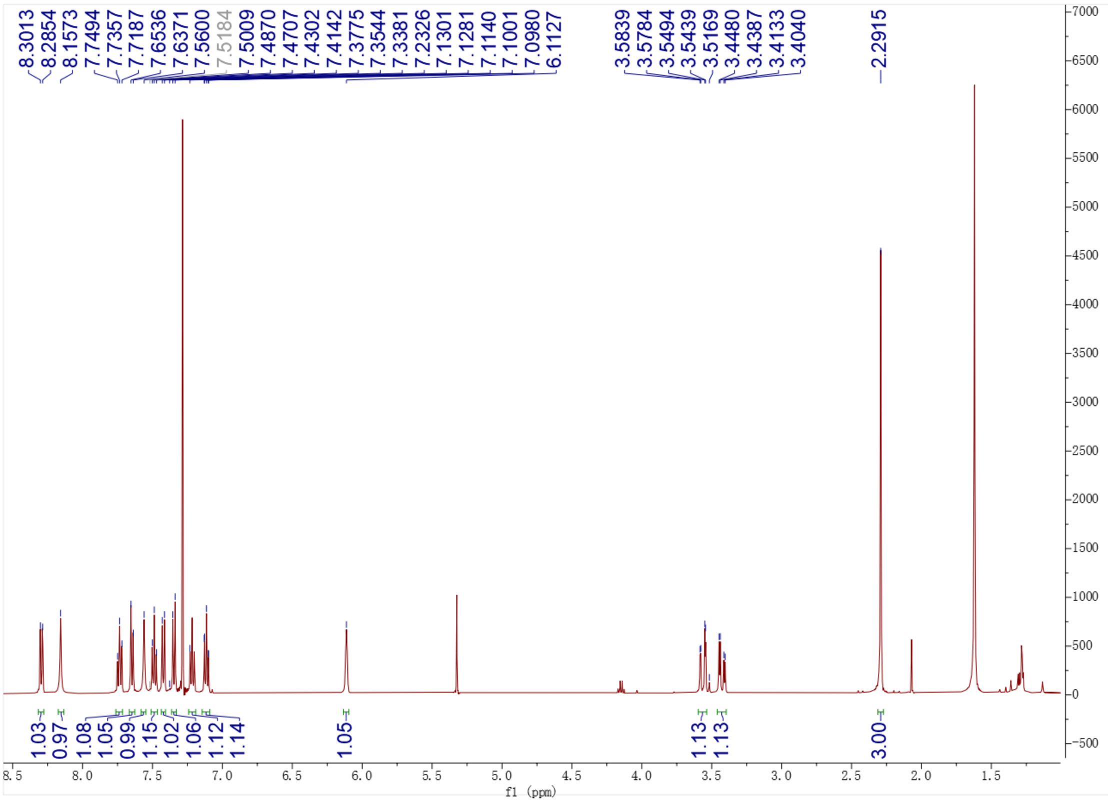

Figure S33. ^{1}H NMR spectrum (500 MHz, $CDCl_3$) of compound **13**.

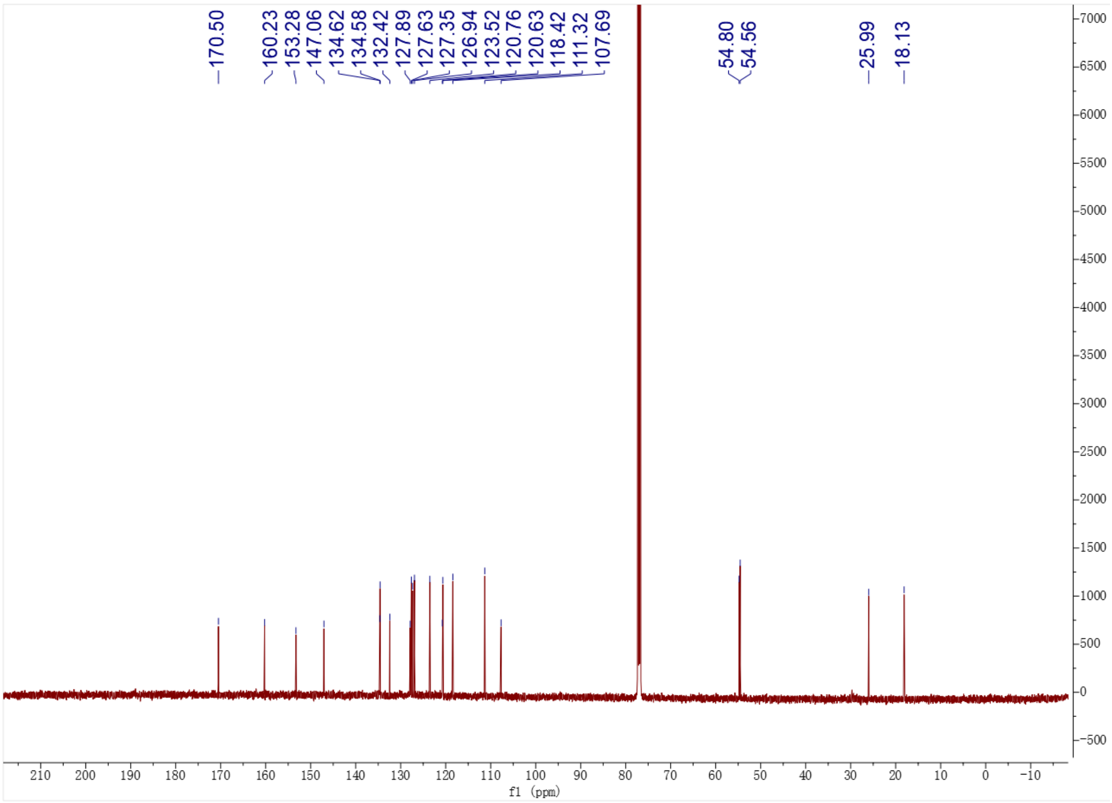

Figure S34. ^{13}C NMR spectrum (125 MHz, $CDCl_3$) of compound **13**.

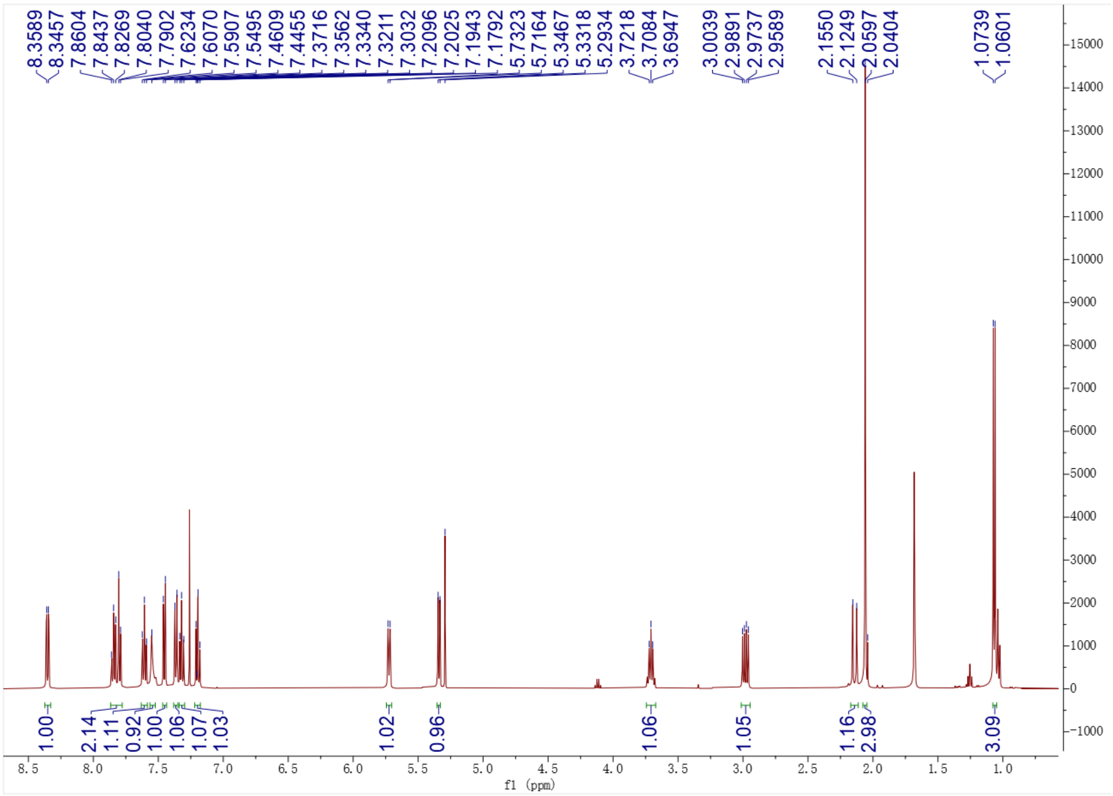

Figure S35. ^{1}H NMR spectrum (500 MHz, $CDCl_3$) of compound **14**.

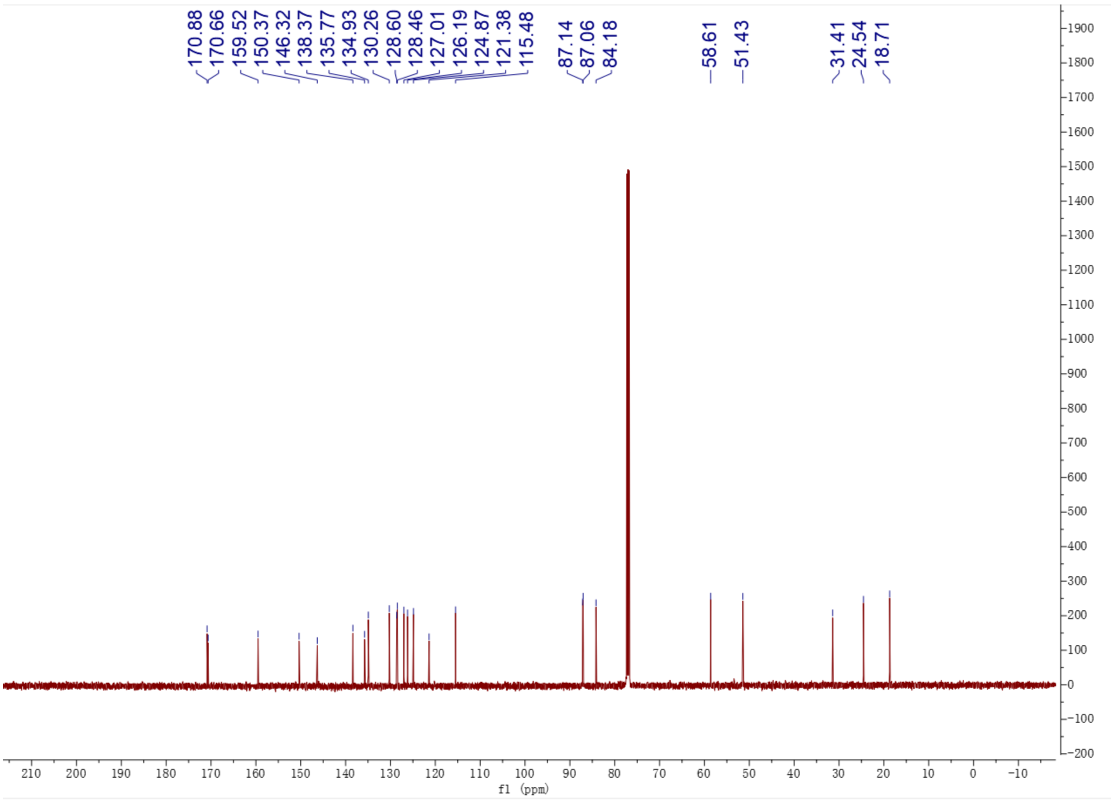

Figure S36. ^{13}C NMR spectrum (125 MHz, $CDCl_3$) of compound **14**.

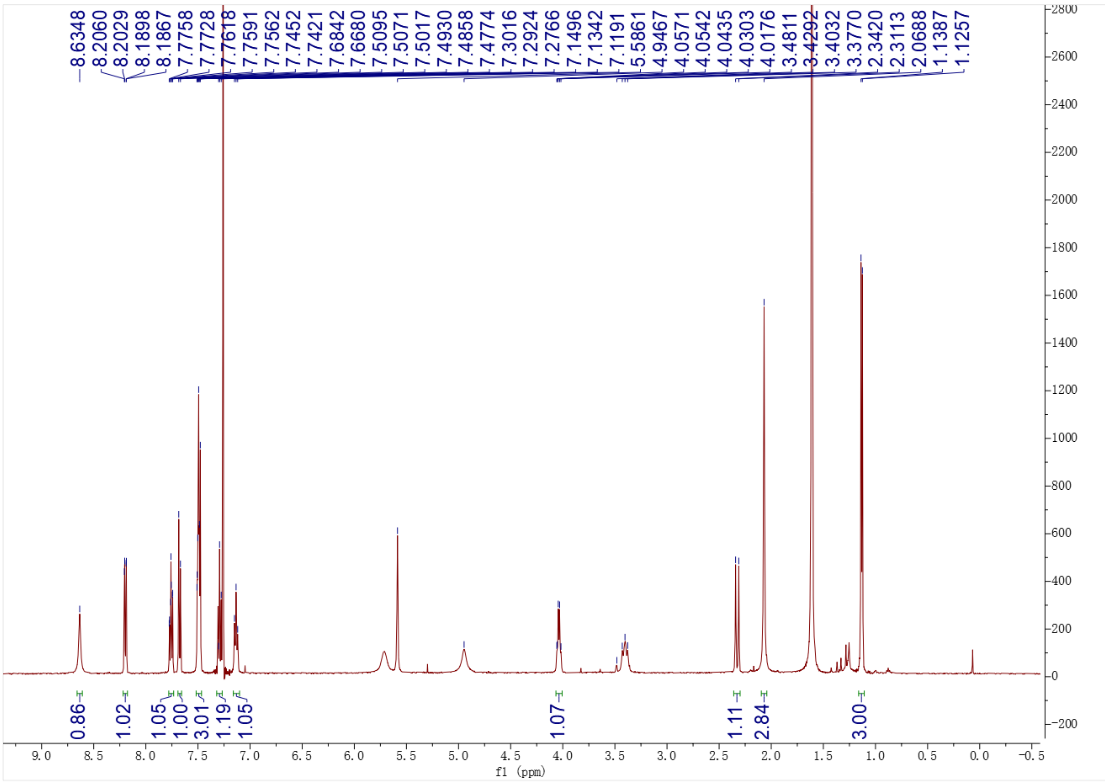

Figure S37. ^{1}H NMR spectrum (500 MHz, $CDCl_3$) of compound **15**.

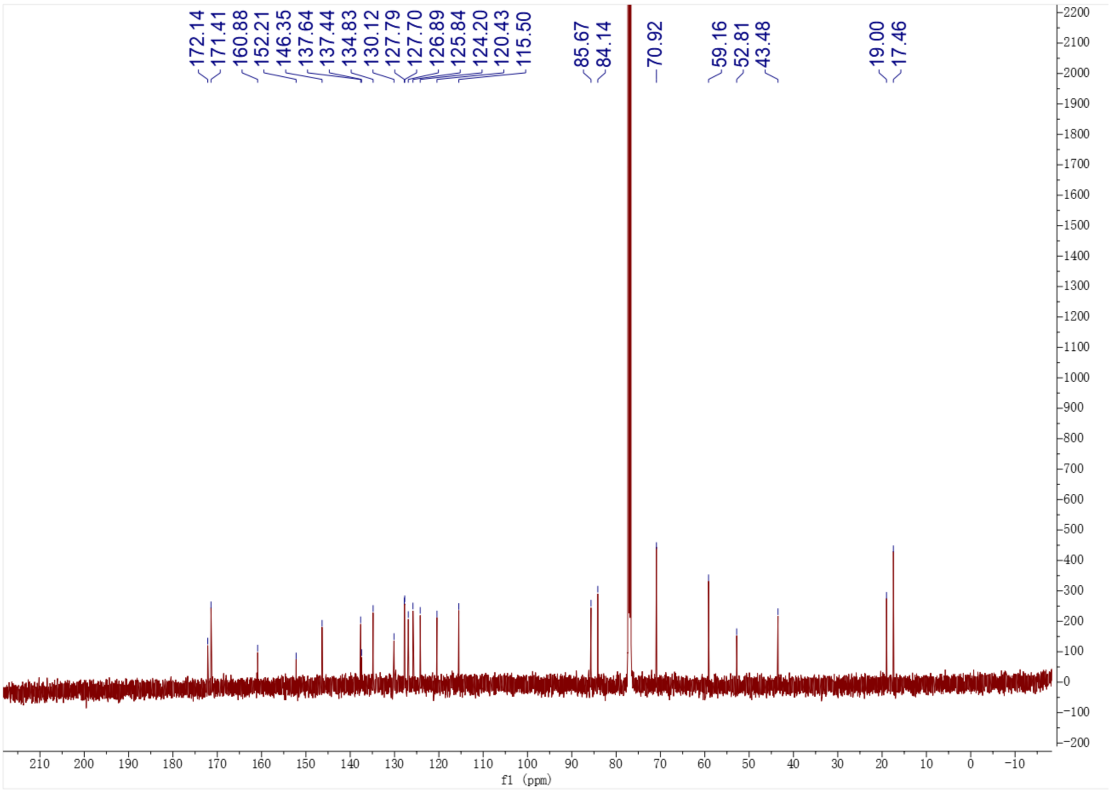

Figure S38. ^{13}C NMR spectrum (125 MHz, $CDCl_3$) of compound **15**.

Table S1. Evaluation of antibacterial activity of compounds **1-15**.

Sample name/number	MIC (μg/mL)		
	**MRSA**	**SA**	***E. Coli***
**Vancomycin**	**0.625**	**0.312**	/
**Polymyxin B**	/	/	**2.5**
**1**	/	/	/
**2**	/	/	/
**3**	**20**	**20**	/
**4**	**3.12**	**3.12**	/
**5**	/	/	/
**6**	/	/	/
**7**	/	/	/
**8**	/	/	/
**9**	/	/	/
**10**	/	/	/
**11**	/	/	/
**12**	/	/	/
**13**	/	/	/
**14**	/	/	/
**15**	/	/	/

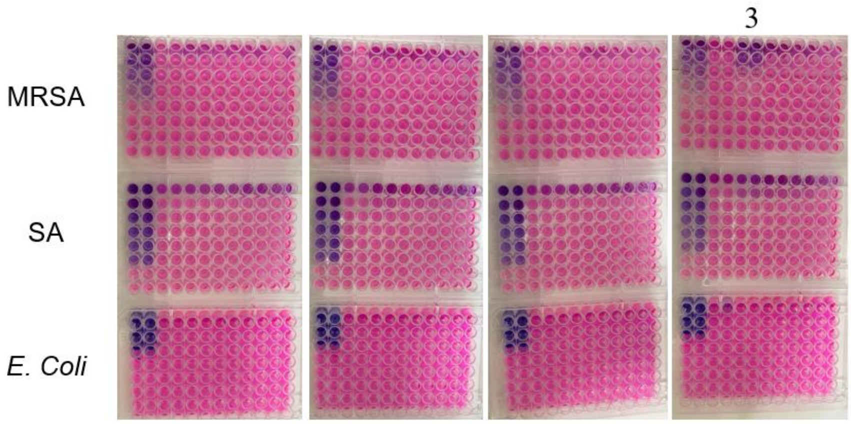

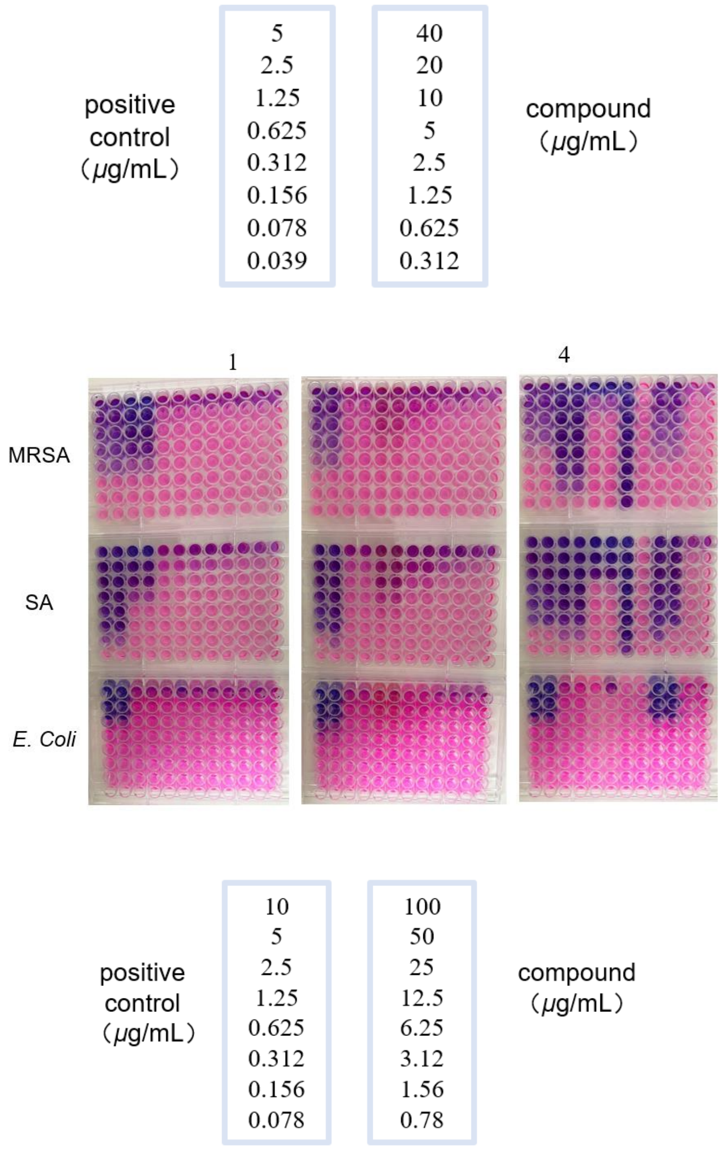

Figure S39. 96-well plate antibacterial results.

Table S2. Evaluation of antibacterial activity of compounds **4**.

Bacterial	G^+/G^-	Helvolic acid	Vancomycin	Polymyxin B
VSE(No.160119481)	G^+	25	0.312	/
VRE(No.151458137)	G^+	12.5	＞10	/
S.dysenteriae(CMCC 51252)	G^-	100	/	2.5
